# Association of Simulated COVID-19 Policy Responses for Social Restrictions and Lockdowns With Health-Adjusted Life-Years and Costs in Victoria, Australia

**DOI:** 10.1001/jamahealthforum.2021.1749

**Published:** 2021-07-30

**Authors:** Tony Blakely, Jason Thompson, Laxman Bablani, Patrick Andersen, Driss Ait Ouakrim, Natalie Carvalho, Patrick Abraham, Marie-Anne Boujaoude, Ameera Katar, Edifofon Akpan, Nick Wilson, Mark Stevenson

**Affiliations:** 1 Population Interventions Unit, Centre for Epidemiology and Biostatistics Research, Melbourne School of Population and Global Health, University of Melbourne, Parkville, Victoria, Australia; 2 Transport, Health and Urban Design Research Lab (THUD), Melbourne School of Design, University of Melbourne, Parkville, Victoria, Australia; 3 Health Economics Unit, Centre for Health Policy, Melbourne School of Population and Global Health, University of Melbourne, Parkville, Victoria, Australia; 4 Burden of Disease Epidemiology, Equity and Cost-Effectiveness Programme (BODE^3^), Department of Public Health, University of Otago, Wellington, New Zealand

## Abstract

**Importance:**

Countries have varied enormously in how they have responded to the COVID-19 pandemic, ranging from elimination strategies (eg, Australia, New Zealand, Taiwan) to tight suppression (not aiming for elimination but rather to keep infection rates low [eg, South Korea]) to loose suppression (eg, Europe, United States) to virtually unmitigated (eg, Brazil, India). Weighing the best option, based on health and economic consequences due to lockdowns, is necessary.

**Objective:**

To determine the optimal policy response, using a net monetary benefit (NMB) approach, for policies ranging from aggressive elimination and moderate elimination to tight suppression (aiming for 1-5 cases per million per day) and loose suppression (5-25 cases per million per day).

**Design, Setting, and Participants:**

Using governmental data from the state of Victoria, Australia, and other collected data, 2 simulation models in series were conducted of all residents (population, 6.4 million) for SARS-CoV-2 infections for 1 year from September 1, 2020. An agent-based model (ABM) was used to estimate daily SARS-CoV-2 infection rates and time in 5 stages of social restrictions (stages 1, 1b, 2, 3, and 4) for 4 policy response settings (aggressive elimination, moderate elimination, tight suppression, and loose suppression), and a proportional multistate life table (PMSLT) model was used to estimate health-adjusted life-years (HALYs) associated with COVID-19 and costs (health systems and health system plus gross domestic product [GDP]). The ABM is a generic COVID-19 model of 2500 agents, or simulants, that was scaled up to the population of interest. Models were specified with data from 2019 (eg, epidemiological data in the PMSLT model) and 2020 (eg, epidemiological and cost consequences of COVID-19). The NMB of each policy option at varying willingness to pay (WTP) per HALY was calculated: NMB = HALYs × WTP − cost. The estimated most cost-effective (optimal) policy response was that with the highest NMB.

**Main Outcome and Measures:**

Estimated SARS-CoV-2 infection rates, time under 5 stages of restrictions, HALYs, health expenditure, and GDP losses.

**Results:**

In 100 runs of both the ABM and PMSLT models for each of the 4 policy responses, 31.0% of SARS-CoV-2 infections, 56.5% of hospitalizations, and 84.6% of deaths occurred among those 60 years and older. Aggressive elimination was associated with the highest percentage of days with the lowest level of restrictions (median, 31.7%; 90% simulation interval [SI], 6.6%-64.4%). However, days in hard lockdown were similar across all 4 strategies. The HALY losses (compared with a scenario without COVID-19) were similar for aggressive elimination (median, 286 HALYs; 90% SI, 219-389 HALYs) and moderate elimination (median, 314 HALYs; 90% SI, 228-413 HALYs), and nearly 8 and 40 times higher for tight suppression and loose suppression, respectively. The median GDP loss was least for moderate elimination (median, $41.7 billion; 90% SI, $29.0-$63.6 billion), but there was substantial overlap in simulation intervals between the 4 strategies. From a health system perspective, aggressive elimination was optimal in 64% of simulations above a WTP of $15 000 per HALY, followed by moderate elimination in 35% of simulations. Moderate elimination was optimal from a GDP perspective in half of the simulations, followed by aggressive elimination in a quarter.

**Conclusions and Relevance:**

In this simulation modeling economic evaluation of estimated SARS-CoV-infection rates, time under 5 stages of restrictions, HALYs, health expenditure, and GDP losses in Victoria, Australia, an elimination strategy was associated with the least health losses and usually the fewest GDP losses.

## Key Points

**Question:**

What has the least health losses and is the most cost-effective of 4 policy responses to the COVID-19 pandemic (aggressive elimination, moderate elimination, tight suppression, and loose suppression) in the state of Victoria, Australia?

**Findings:**

In this simulation modeling economic evaluation of health losses and costs from COVID-19 policy responses, aggressive elimination was the most cost-effective from a health system perspective in 64% of simulations above a willingness to pay of $15 000 per health-adjusted life-years, followed by moderate elimination in 35% of simulations. Moderate elimination was most cost-effective from a gross domestic product (GDP) perspective (ie, including GDP losses in addition to health expenditure) in half of the simulations, followed by aggressive elimination in a quarter.

**Meaning:**

While there is considerable uncertainty in outcomes for all 4 policy responses, the 2 elimination options appear to be the most optimal from both health system and health plus GDP perspectives.

## Teaser

This economic evaluation determines the optimal policy response to the COVID-19 pandemic in Victoria, Australia, using a net monetary benefit approach for policies ranging from aggressive elimination and moderate elimination to tight suppression and loose suppression.

## Introduction

There is no best approach for all countries to follow when handling the COVID-19 pandemic. Rather, each country will devise an approach given updated scientific understanding, its infection load, its ability to manage borders and quarantine, vulnerability and age structure of its population, health system capacity, economic and other resources, social values and preferences,^1^ and, more recently, timelines and progress to vaccination. One way to support this challenging decision-making is to use an integrated assessment of health and economic outcomes. Such an optimization approach is often implicit in commentaries, with phrases such as ensuring the cure is not worse than the disease.^2^ But seldom is this balance or optimization explicitly defined and empirically addressed.

To this point in the pandemic, a standard cost-effectiveness analysis resting on epidemiological modeling has not been widely used with the exception of cost-effectiveness and cost-benefit studies looking at a narrow range of policy measures, such as social distancing, treatments, or a vaccine, or focused on a particular population group, such as testing strategies in the residential college campus setting.^3^ Existing reviews^4^ show a mix of methodologies evaluating a range of interventions, with studies evaluating broader policy measures among a wider population^5^ being less common. Undertaking cost-effectiveness studies in a pandemic is challenging for reasons such as which perspective to use (eg, health system only or societal) and uncertainty in many inputs—yet, be it implicit or explicit, cost-effectiveness does feature in decision-making.

In this epidemiologic and economic evaluation, we applied an integrated epidemiological and economic modeling approach to estimate the COVID-19 pandemic control strategy associated with the highest net monetary benefit (ie, most cost-effective, hereafter *optimal*) for varying willingness to pay (WTP) per health-adjusted life-year (HALY) thresholds in a single high-income jurisdiction: the state of Victoria, Australia, as it was coming out of its second COVID-19 wave starting September 1, 2020. The SARS-CoV-2 infection rates in response to policy options were modeled with an agent-based model (ABM)^6^ that was used successfully to chart Victoria’s road map out of its second COVID-19 wave to eliminate community transmission; the infection rates were then inputted to a proportional multistate lifetable model (PMSLT)^7^ to estimate health costs. The 4 policy options included continuing stringent social and economic controls in an effort to achieve elimination of local transmission: (1) aggressive elimination (eg, New Zealand and Victoria in 2020 and China after the outbreak in Wuhan), (2) moderate elimination (eg, New South Wales, Australia, which went later into lockdown and earlier out of lockdown than the state of Victoria and New Zealand to manage an outbreak, but still with a goal of elimination), (3) tight suppression (eg, some East Asian countries, including South Korea and Singapore, which had low daily rates that the public health workforce could manage with contract tracing), and (4) loose suppression (eg, countries including Spain, the United Kingdom, and France, which had daily rates within health-services capacity between their first and second COVID-19 waves). We used both a health-system perspective and a gross domestic product (GDP) perspective (the latter adding GDP losses to health expenditure).

## Methods

### Conceptualization and Specification of Policy Scenarios

Most countries or jurisdictions have some form of stages, tiers, or levels of control measures that escalate or de-escalate depending on SARS-CoV-2 infection rates. eTable 1 in the Supplement summarizes the 5 stages applied in this study in conceptual terms, and eTable 4 in the Supplement summarizes how these stages were coded in the ABM. For example, Stage 1 has no restrictions and moderate mask use in indoor settings, and Stage 1b sees 25% of workers with restricted movements (eg, working from home). Stage 2 sees 50% of workers with restricted movements, and Stage 3 sees this increasing further to 75% and with most schools closed. Stage 4, hard lockdown, has all schools closed, marked reductions in all agents’ (ie, simulants in the model) distance of movement, and only 20% of adults considered essential workers (and able to move more freely). We assume that mandatory mass mask wearing will be retained in stages 2 to 4, as well as in stages 1 and 1b in public transport and busy indoor settings where physical distancing is difficult.

The 4 policy responses (aggressive elimination, moderate elimination, tight suppression, and loose suppression) all have the same stages but different rules encoded in the ABM to escalate and de-escalate stages, as summarized in eTable 2 in the Supplement and, for tight suppression (as 1 example), in eFigure 1 in the Supplement. Essentially, the elimination strategies have little tolerance for cases and activate escalating stages of restriction rapidly as case numbers increase in an attempt to eliminate community transmission, whereas the 2 suppression strategies do not aim to eliminate transmission (although it may happen by chance) but rather aim to keep infection rates between 1 to 5 cases per million per day for tight suppression and 5 to 25 cases per million per day for loose suppression.

### Agent-Based Model

Further details on the ABM specification, validation, and calibration are given elsewhere.^6,8^ Briefly, the model is a generic COVID-19 model of 2500 agents that is then scaled up to the population of interest (the state of Victoria has a population of 6.4 million). The ABM has a daily cycle length. Each agent (an individual simulant) moves in a 2-dimensional space, contacting other agents, with opportunities for between-agent infection given transmission rates and contact duration. The model was calibrated to the first COVID-19 waves in New Zealand and Australia, has performed well in prediction (noting inherent stochastic uncertainty),^6^ and has been used by the Victorian government to develop its road map out of the second COVID-19 wave in Victoria.^9,10^ eTables 2, 3, and 4 in the Supplement provide the parameterization of stages in the ABM. The simulation was for 1 year, from September 1, 2020 (and 6 and 18 months in sensitivity analyses), equivalent to a 1-year intervention duration.

### GDP and Unemployment Estimates

Each stage was associated with a GDP consequence per week compared with business as usual or no COVID-19 (Stage 1, $0.54 billion; Stage 1b, $0.60 billion; Stage 2, $0.73 billion; Stage 3, $1.28 billion; and Stage 4, $2.61 billion). See eTable 9 and eAppendix 1 in the Supplement for details.

### Proportional Multistate Life Table Model

A PMSLT model^7,11^ consists of parallel cohort life tables for men and women by 5-year age cohort in 2020, simulated for mortality, morbidity, and health expenditure over the remainder of their lives (ie, a lifetime horizon). Beneath this main all-cause life table sit parallel disease life tables that proportions of the cohort reside in based on disease incidence, case fatality, and remission rates (see eTables 11-13 and eFigure 2 of eAppendix 4 of the Supplement). Herein, we use a PMSLT model adapted for COVID-19 with disease states for SARS-CoV-2 infection and (for sensitivity analyses only) 3 indirect consequences of COVID-19 policy responses through changes in GDP and unemployment rates, road traffic crashes (RTCs), depression, and anxiety. The inputs to the PMSLT model are summarized in eTable 5 in the Supplement.

The monthly number of SARS-CoV-2 infections from each of the 100 ABM model runs were distributed by age to match the actual proportionate distribution by age of cases in Victoria (skewed to older ages owing to outbreaks in residential aged care). The outputs of the PMSLT model include incremental HALYs and net health expenditure owing to SARS-CoV-2 infections and average citizen health expenditure among the living, incremental to no COVID-19 or business as usual, using a 3% discount rate^12^ (see eTables 14-18 in eAppendix 4 of the Supplement). Up-front health expenditure (eg, intensive care unit capacity, surveillance systems) was assumed to be an identical sunk cost between scenarios. Among those 60 years and older, 31.0% of SARS-CoV-2 infections, 56.5% of hospitalizations, and 84.6% of deaths occurred.

### Analyses

#### Monte Carlo Analysis

Both the ABM and PMSLT model were each run 100 times for each policy scenario. Within each run, random draws were made from each input parameter’s probability density function (ie, its uncertainty interval; see eTable 3 in the Supplement for uncertainty distributions about input parameters). For the ABM, additional stochastic or individual-level variability was included in each run (eg, variability in a contact leading to acquiring an infection). Thus, there were 100 output values of the number of SARS-CoV-2 infections from the ABM for each policy response option; likewise, there were 100 output values of HALYs and net health expenditure (and GDP costs) from the PMSLT model. The median on these 100 values was reported as the central estimate and the 5th and 95th percentiles as the lower and upper limit of the simulated range of output values.

#### Net Monetary Benefit

We then estimated net monetary benefit within each of the 100 iterations for all 4 scenarios, where net monetary benefit monetizes the HALYs for a range of WTP per HALY thresholds (see eAppendix 2 in the Supplement for the formula used). These outputs can then be shown as cost-effectiveness acceptability curves showing the probability that each scenario has the highest net monetary benefit across the 100 model runs for varying WTP thresholds.

#### Sensitivity Analysis

The HALYs and net health expenditure were re-estimated for 0% and 6% discount rates. The base assumption of an intervention duration (ie, the time to a vaccine) was 12 months; we reran the PMSLT model for 6-month and 18-month duration outputs from the ABM.

The main model only considered SARS-CoV-2 infections. It seems likely that there are associations of social restrictions with mental health (eg, deleterious for depression and anxiety but neutral for suicides) and reduced trauma arising from RTCs (beneficial). Ideally, one would estimate the counterfactual associations of these health events at each level (lockdown stage) of COVID-19 policy response using published natural experiments, but this is a major task with, currently, inadequate data. Therefore, as a simple sensitivity analysis we first, for RTCs, used the association of mobility data with lockdown stages experienced in Victoria and, in turn, the association of mobility data with Victorian RTC rates (see eAppendix 5, eFigures 3 and 4, and eTables 19-21 in the Supplement for additional data). Second, for depression and anxiety we assumed time spent in stages 1, 1b, 2, 3, and 4 had 2%, 4%, 6%, 8%, and 10% increases in the age by sex prevalence of depression and anxiety, respectively. Finally, a key rationale for tight suppression is that by keeping case numbers low, contact tracing is more effective, meaning one can function with lesser social and public health restrictions. We therefore reran the 4 policy scenarios incorporating a dynamic contract tracing variable that varied with the log of the number of cases per day (% of contacts identified per day = 0.586 − ln (cases per day) × 0.06213). Accordingly, the base case of 30% of contacts traced each day was assumed to apply at 100 cases per day, improving to 34% at 50 cases per day, 39% at 25 cases per day, 44% at 10 cases per day, and 50% at 4 cases per day. For additional information on model construction and parameter inputs see eTables 1 to 9 in the Supplement.

Findings are reported in accordance with the Consolidated Health Economic Evaluation Reporting Standards (CHEERS) reporting guideline.^13^ Rapid ethics review was necessary to access Victorian government COVID-19 data, but otherwise all other data inputs we aggregated did not require ethics approval, as determined by the Melbourne School of Population and Global Health Human Ethics Advisory Group.

## Results

### Policy Response Strategies

**Figure 1.**
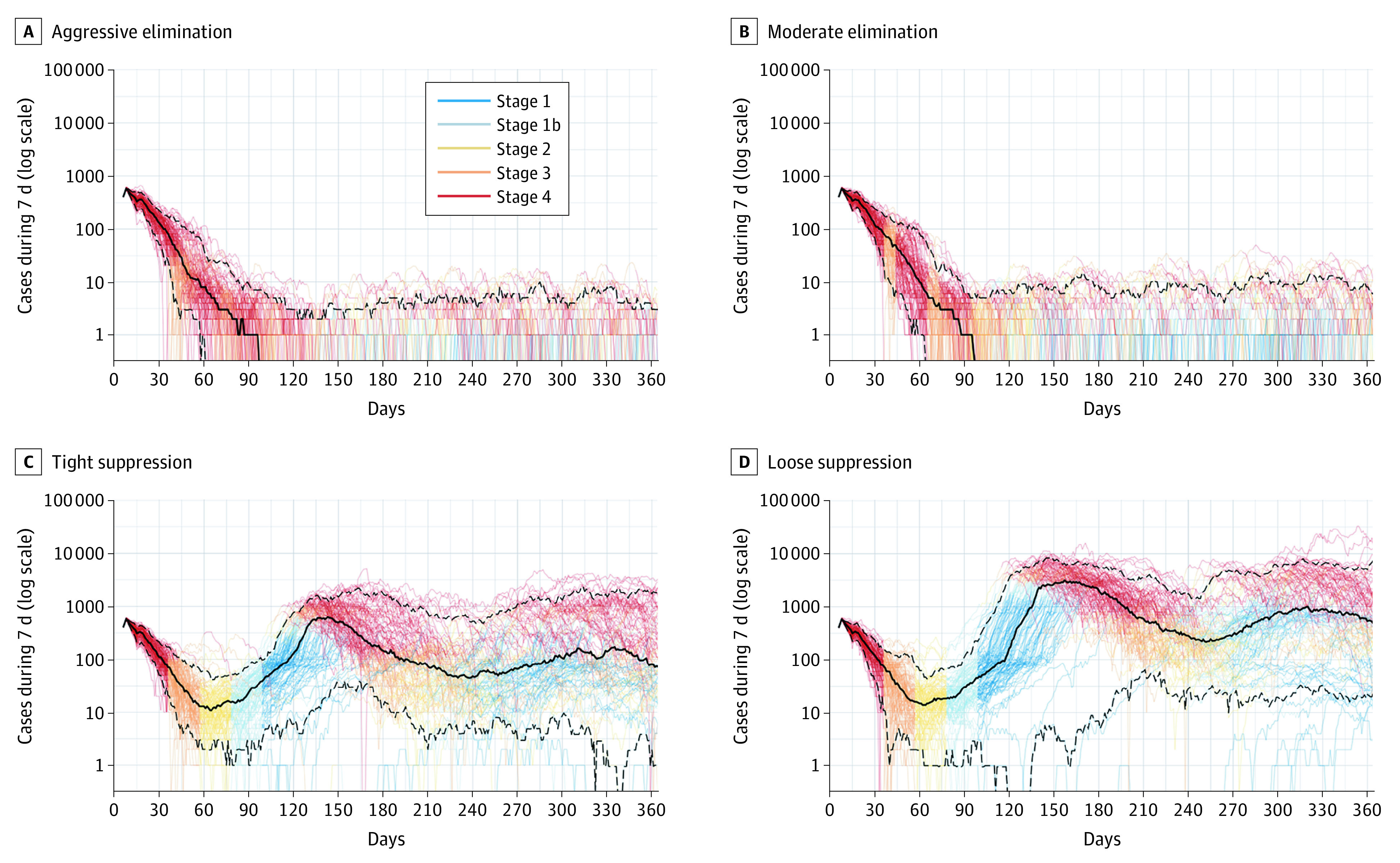
Cases per Day Among the 4 Simulated Policy Responses to the COVID-19 Pandemic Traces are for each of 100 agent-based model iterations, with coloring indicating the stage of restrictions active over time for each iteration. The solid black lines indicate the medians across 100 iterations; dashed black lines, 5th and 95th percentiles across 100 iterations.

The daily estimated numbers of SARS-CoV-2 infections for 100 runs of each strategy are shown in Figure 1. All strategies were associated with a decrease in the first 2 months, then marked stochastic variation in daily numbers (hence varying times in stages 1-4, as shown in Figure 1 by varying the color of each run’s trace, with red as Stage 4 [hard lockdown] and orange as Stage 3 [soft lockdown]).

**Figure 2.**
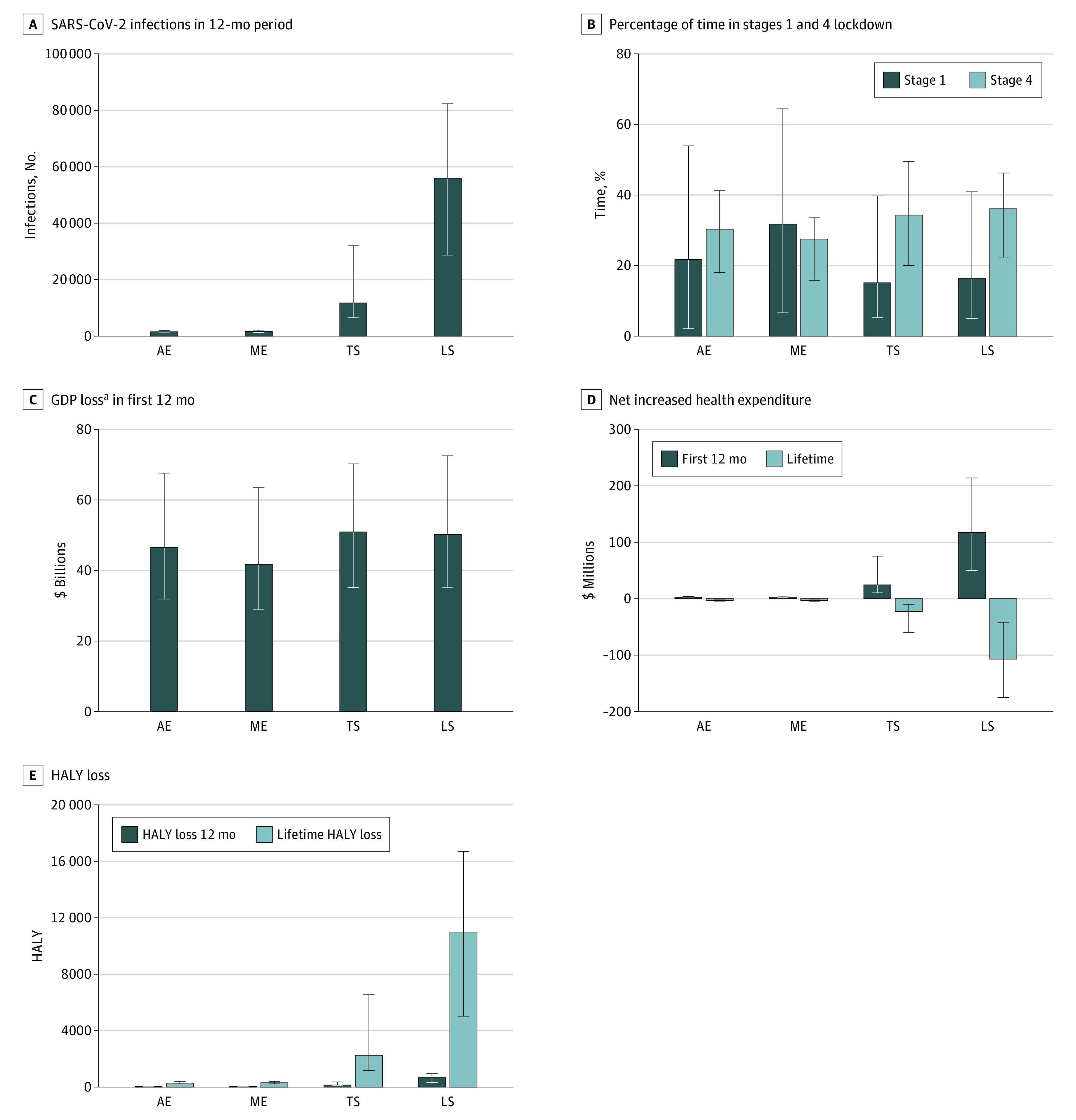
SARS-CoV-2 Infections, Time in Stages of Lockdown, GDP Loss, Net Health Expenditure, and HALY Loss Among the 4 Simulated Policy Responses to the COVID-19 Pandemic All data are incremental to pre–COVID-19 business as usual. Data used to populate these figures are shown in eTables 6 and 7 in the Supplement. Error bars indicate 90% simulation intervals. AE indicates aggressive elimination; GDP, gross domestic product; HALY, health-adjusted life-years; LS, loose suppression; ME, moderate elimination; and TS, tight suppression. ^a^Losses are before factoring in the (large) economic stimulus packages; see footnote to eTable 6 in the Supplement for details.

Figure 2 shows the outputs from the ABM in terms of estimated numbers of infections and numbers of days at varying policy stringency (tabular data for these and more analyses are summarized in eTables 6 and 7 in the Supplement). The cumulative number of additional SARS-CoV-2 infections from the starting date over 1 year were lowest for aggressive elimination (median, 1530; 90% simulation interval [SI], 1150-2030). Infections were highest at 55 900 (90% SI, 28 700-82 300) for loose suppression.

Moderate elimination (median, 31.7%; 90% SI, 6.6%-64.4%) was associated with the greatest percentage of days with the least restrictions (Stage 1) compared with 21.7% (90% SI, 2.1%-53.9%) for aggressive suppression, 15.1% (90% SI, 5.3%-39.7%) for tight suppression, and 16.3% (90% SI, 5.0%-40.9%) for loose suppression. However, the percentage of days with maximum restrictions (Stage 4) was similar across all strategies (medians ranging from 27.5% [90% SI, 15.8%-33.7%] for moderate elimination to 36.1% [90% SI, 22.4%-46.2%] for loose suppression). Consequently, given much of the total loss of GDP was associated with Stage 4, while moderate elimination was associated with the lowest median GDP loss up to 12 months of $41.7 billion (90% SI, $29.0-$63.6 billion), it was similar across strategies when allowing for the wide SIs.

eTable 6 in the Supplement demonstrates that estimated deaths varied by stage pro rata with SARS-CoV-2 infections. The crude case fatality rate was estimated to be about 4% across scenarios, reflecting the concentration of cases in residential aged care during the Victorian second COVID-19 wave. Lifetime HALY losses (Figure 2), discounted at 3% annually, were similar for aggressive elimination (286 HALYs; 90% SI, 219-389 HALYs) and moderate elimination (314 HALYs; 90% SI, 228-413 HALYs), and 7.5 and 36 times higher for tight suppression and loose suppression, respectively (eTable 7 in the Supplement). Net health expenditure differences compared with business as usual varied over the time horizon, with expenditure in the first year increasing by $2.71 million (90% SI, $1.49-$3.82 million) for aggressive elimination and up to $117 million (90% SI, $50-$214 million) for loose suppression. However, in out-years net health expenditure decreases compared with business as usual in all strategies owing to fewer people being alive and incurring increasing health expenditure as they age. eAppendix 3 and eTable 10 in the Supplement provide more information on health expenditure.

### Differences Between Policy Responses

The Table summarizes the differences between policy response options, for the median and percentiles of the differences within each of the 100 runs. There were clear differences in health loss with no overlap in 90% SIs for the 2 elimination options compared with the 2 suppression options.

**Table.** Differences in Main Outputs Among the Simulated Policy Responses to the COVID-19 Pandemic for a 12-Month Intervention Period^a^

Strategy	Median (90% SI)			
	Aggressive elimination	Moderate elimination	Tight Suppression	Loose suppression
**Compared with aggressive elimination**				
HALY loss over lifetime	0 [Reference]	21 (−90 to 129)	1927 (871 to 6249)	10 661 (4776 to 16 396)
Net health expenditure over lifetime, $ million	0 [Reference]	−0.2 (−1.3 to 0.9)	−19.2 (−57.4 to −7.4)	−103.2 (−172.8 to −40.2)
Estimated GDP loss, $ billion	0 [Reference]	−5.2 (−18.6 to 11.0)	3.3 (−12.1 to 23.6)	4.3 (−15.9 to 19.4)
**Probability of being largest across policies, %**				
HALY loss over lifetime	0	0	7	93
Net health expenditure over lifetime	62	37	0	1
Estimated GDP loss	19	4	33	44
**Probability of being smallest across policies, %**				
HALY loss over lifetime	63	36	0	1
Net health expenditure over lifetime	0	0	7	93
Estimated GDP loss	23	49	14	14

Abbreviations: GDP, gross domestic product; HALY, health-adjusted life-year; SI, simulation interval.

^a^ 90% SIs of the differences were calculated within each run, ensuring each within-run comparison was subject only to stochastic variability and proportional multistate life table model input parameter uncertainty.

There was considerable stochasticity and overlap between policies in estimated GDP loss associated with the elimination strategies that use stage 3 and 4 restrictions (where GDP loss compared with business as usual is maximal) more readily and early. Meanwhile, the suppression strategies have to move into stages 3 and 4 frequently to keep infection and case rates within the target range. The net result is widely overlapping 90% SIs in GDP loss for the 4 strategies (Table). Loose suppression was associated with the greatest GDP loss in 44% of simulations, followed by tight suppression at 33% and aggressive elimination at 19% (Table). The percentage of runs in which GDP loss was the least occurred in moderate elimination (49%), followed by aggressive elimination (23%) and both suppression options (14% each) (Table).

**Figure 3.**
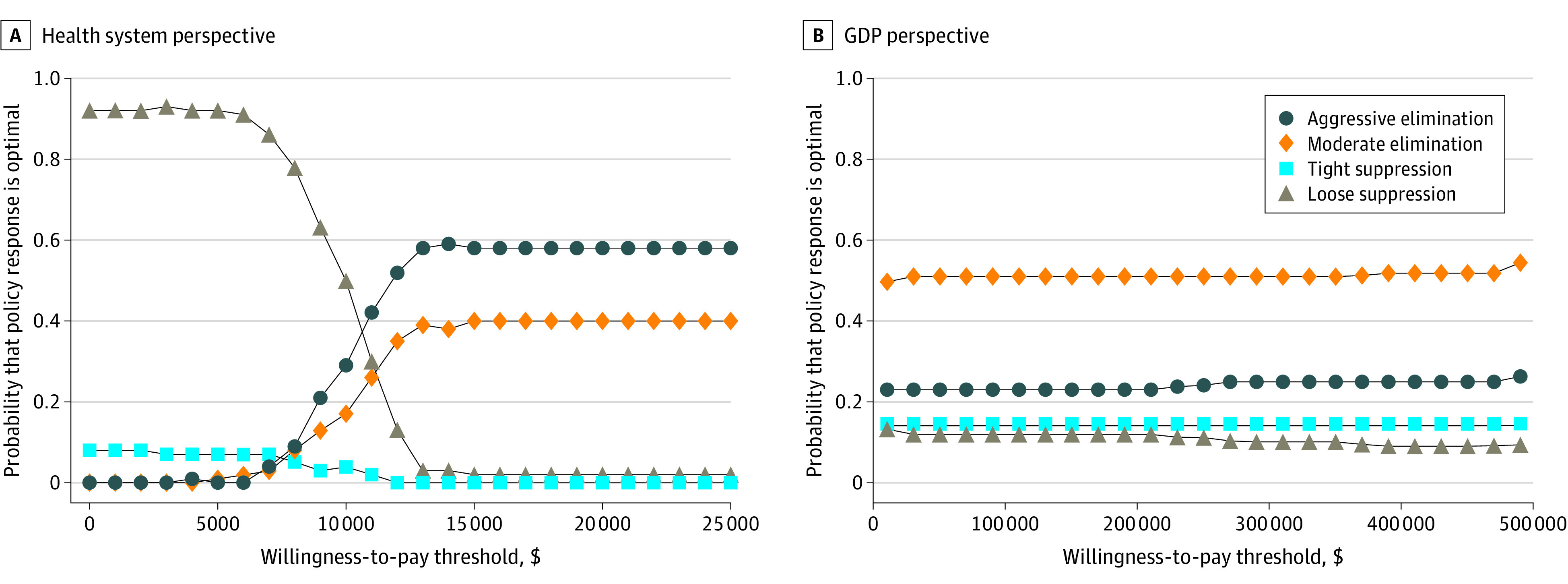
Cost-effectiveness Acceptability Curves Among the 4 Simulated Policy Responses to the COVID-19 Pandemic GDP indicates gross domestic product.

Figure 3 shows the cost-effectiveness acceptability curves for the 4 strategies. Interventions tended to be considered cost-effective up to approximately GDP per capita per HALY in high-income countries, which is about $55 000 for Australia. For WTP greater than $15 000 per HALY, aggressive elimination was estimated to be optimal in 64% of simulations, followed by moderate elimination in 35% of simulations.

From a GDP perspective, moderate elimination was estimated to be optimal for half of the simulations regardless of WTP, and aggressive elimination was estimated to be optimal one-quarter of the time. However, there was a 10% to 15% probability of either tight suppression or loose suppression being estimated to be optimal, highlighting uncertainty in estimates.

### Sensitivity Analyses

**Figure 4.**
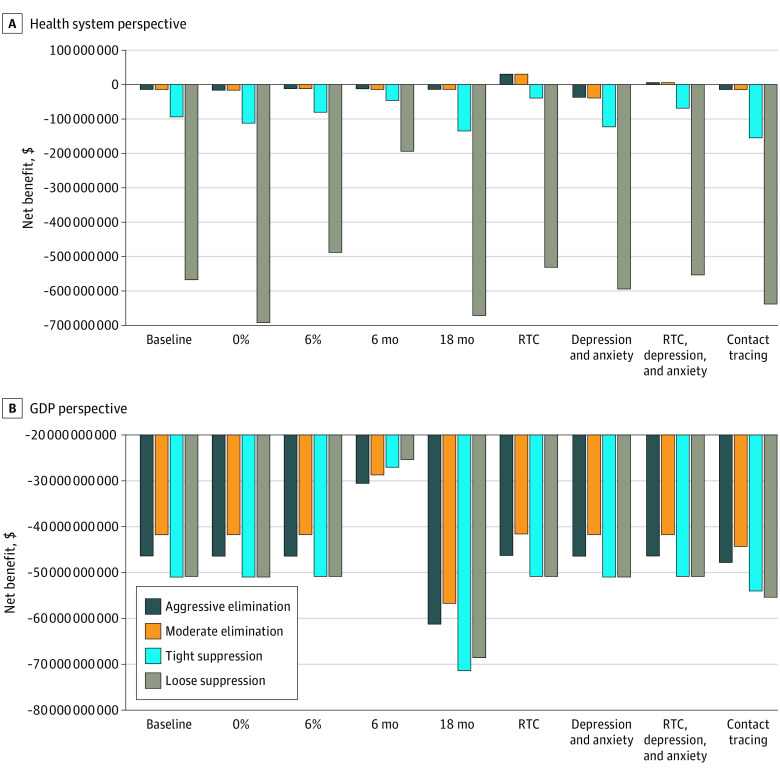
Net Monetary Benefit at Per-Capita GDP ($55 000) Using Median Expected Values and Lifetime Time Horizon Tabular data for these figures are presented in eTable 8 in the Supplement. GDP indicates gross domestic product; RTC, road traffic crash.

Results for the sensitivity analyses are shown in Figure 4. These analyses were conducted using the median number of infections from the ABM propagated through the PMSLT model as 1 expected or median value run and for a WTP of $55 000 per HALY (ie, Australia’s GDP per capita) (Monte Carlo simulation was not feasible for sensitivity analyses due to long model run times). This highlights that COVID-19 policy responses, using a non–COVID-19 comparator, are about choosing the least worst option given the large negative estimates. From a health system cost perspective, the 2 elimination strategies were estimated to be optimal across all sensitivity analyses, and the loose suppression strategy as clearly the worst. From a GDP perspective, there was less relative separation in the policy options, but moderate elimination is always optimal. The exception is when the intervention is only 6 months in duration; here, loose suppression is optimal. This scenario might reflect an early end to the pandemic owing to, for example, a rapid and successful vaccination program.

Including RTC in the model demonstrated that health losses were associated with health gains of 661 and 684 HALYs for aggressive elimination and moderate elimination, respectively (eTable 8 in the Supplement). Conversely, including depression and anxiety (but not RTC) was associated with health loss increases of 738 and 762 HALYs, respectively. Noting the speculative nature of these disease scenario analyses, adding RTC, depression, and anxiety was associated with modest health gains in the 2 elimination strategies owing to RTC decreases tipping the balance. There was little relative association with health losses for loose suppression owing to COVID-19–related health loss being much larger than any countervailing force of RTC.

## Discussion

This simulated modeling study estimates the health and economic outcomes associated with a range of COVID-19 policy response strategies. There are several important findings. First, there was large stochastic variation in SARS-CoV-2 infections between runs of the same strategy (Figure 1), meaning that an optimal strategy, on average, may not always be optimal in practice. Second, there was little difference in the median GDP loss across strategies, as elimination strategies use stage 3 and 4 lockdowns at lower thresholds, whereas suppression strategies are forced to use stage 3 and 4 lockdowns to keep infections in the target range. Furthermore, the GDP findings are very uncertain. However, when comparing across countries, Baker et al^14^ suggest that GDP loss during 2020 as estimated by the International Monetary Fund has been lower in countries using elimination as opposed to suppression strategies. Loose suppression in the present study had the largest probability (44%) of having the highest GDP loss, tight suppression had a 33% probability, and aggressive elimination still had a 19% probability of the highest GDP loss. Third, the strategy with the highest net monetary benefit (hence, *optimal* on cost-effectiveness grounds) was aggressive elimination from a health system perspective and moderate elimination from a GDP perspective. Fourth, reducing the intervention duration from 12 to 6 months (reflecting a scenario in which most people are vaccinated within 6 months) saw loose suppression displace moderate elimination as optimal from a GDP perspective—offering some support for countries with high rates (eg, United Kingdom, United States, Spain, France) to ride it out with short lockdowns to keep infection rates in check until vaccination coverage is high.

Compared with the scant cost-effectiveness literature on COVID-19 policy responses so far,^3,4^ findings of this study offer improvements for consideration. First, to the best of our knowledge, this study is the only one to use an ABM to simulate dynamic policy regimes that move up and down stages based on triggers of daily case numbers, capturing the large stochastic variation in how case numbers evolve. Other model frameworks use rapid approaches,^15^ decision tree and Markov models, or a variation of a dynamic (susceptible-infected-recovered style) model, which allow for only limited complexity. Second, sensitivity analyses around discount rates and diseases to include do not supplant the main conclusions as to which policy is optimal. Next, the consideration of both health costs and GDP consequences associated with enacted social policies take us beyond a limited health sector perspective.^16^ A previous Australian analysis^17^ has also found that elimination may be better for both health and economic outcomes but was not as sophisticated as the present study in allowing for dynamic policy settings, other disease sequalae, and viral incursion owing to quarantine breaches. Interestingly, the present results echo somewhat a US study finding that social distancing may be a cost-effective strategy relative to herd immunity if an effective therapy or vaccine can be introduced within a reasonable (<12 months) time frame.^14^ The eReferences in the Supplement provide more sources on this topic.

### Limitations

This study design is a simulation analysis, not an observation of how different policy responses played out in exactly the same circumstances (ie, a highly improbable randomized trial or a natural experiment where 2 jurisdictions could be found that were identical, or exchangeable, other than the policy applied). However, given the highly stochastic nature of COVID-19 transmission and epidemics (eg, the traces for iterations in the ABM in Figure 1), even such an ideal study design would not give one good accuracy predicting the future. Thus, simulating many versions of what might happen (ie, the Monte Carlo simulations) is necessary to generate a range of expected outcomes and their uncertainty.

Second, as to the generalizability of the present study to non-Australasian settings, we have deliberately attempted to conceptualize the tight suppression scenario to approximate countries such as Singapore and South Korea, and the loose suppression scenario to approximate regions such as Europe (before Christmas 2020 when control was lost). Nevertheless, caution must be exercised in transferring the present findings to other countries, especially with differences in demographic profiles (eg, younger populations), the advent of new COVID-19 variants of concern, and vaccination, which we are currently incorporating in future Australasian and Pakistan modeling.

This study also forces us to confront the reality that the outcomes of COVID-19 policy responses are very uncertain owing to inherent stochastic uncertainty (ie, chance occurrences of who contacts whom, leading to highly variable epidemic curves) and uncertain knowledge about the effectiveness of, and compliance with, behavioral infection management and other social interventions. While stochastic uncertainty will persist despite improved knowledge, there are other sources of uncertainty that may reduce. First, contact tracing is improving as the pandemic progresses, which will favor elimination strategies assuming that contact tracing is most effective when caseloads are low. Second, it is strongly suspected (but hard to quantify) that unintended consequences of policy responses on non–COVID-19 health outcomes and long COVID-19 are important. For example, in plausible sensitivity analyses we used for RTC and depression and anxiety, the net health consequences for elimination strategies with low SARS-CoV-2 caseloads can vary markedly in percentage terms.

## Conclusions

Results of this epidemiologic and economic evaluation study show that while the world is ramping up vaccine rollouts, it will take time. This leaves populations vulnerable and future resurgences of infections, morbidity, and mortality likely. This study suggests that an elimination strategy was estimated to be optimal in 2020 for countries that had the opportunity to pursue or maintain that approach. Going forward, given global eradication of SARS-CoV-2 is unlikely, countries that currently have (near) zero community transmission will need to pivot to opening their borders at some point in the future. The question of when to open was beyond the scope of the current study and calls for further modeling that can assist policy makers to make the best decisions. However, extrapolating from the findings in this study, maintaining something like an elimination approach until herd immunity (or approaching it) is achieved through vaccination will most likely be optimal.

## Notes

Supplement. eTable 1. Physical distancing and other measures by ‘stage’ as used in Victoria, Australia, for control of COVID-19 eTable 2. Conceptualisation and specification of the triggers to shift between stages by policy scenario eTable 3. Parameter estimates and ‘agent’ characteristics most relevant to current paper used in the agent-based model (for full details see source code and ODD protocol in footnote to this table) eTable 4. Key input parameters by level of policy stringency in the ABM eTable 5. Input parameters to PMSLT (excluding those from ABM) and GDP costs (inputs in italics only used in sensitivity analyses) eTable 6. Outputs from ABM, and estimated GDP loss, for ‘best’ scenario: 12 months intervention or ABM time horizon (i.e., assumed vaccination available in 12 months); 1% probability per day of incursion of infected person into Victoria in elimination strategies eTable 7. Estimate incremental health loss (HALYs) loss compared to BAU (i.e., no SARS-CoV-2 pandemic) and additional health expenditure (3% discount rate) eTable 8. Sensitivity analyses incremental health loss (HALYs) compared to BAU (i.e., no SARS-CoV-2 pandemic) and additional health expenditure and GDP loss (3% discount rate; in US$ millions), using the median infection rate across 100 simulations (% in parentheses are change relative to baseline) eFigure 1. Stages and triggers for tight suppression eAppendix 1. Estimates of GDP loss by stage, from Australian and Victorian Treasuries eTable 9. Estimates of per week GDP loss by stage, relative to no restrictions. Scope included GDP losses caused by Victorian restrictions, but borne beyond Victoria eAppendix 2. Net Monetary Benefit eAppendix 3. Average citizen annual health expenditure eTable 10. Per-capita annual health expenditure in Australia, by sex by age (2019 US$) eAppendix 4. SARS-CoV-2 parameters eTable 11. Numbers of confirmed SARS-CoV-2 cases, hospitalisations, ICU admission, and deaths in Victoria, Australia eFigure 2. Ln odds of hospitalisation, ICU admission, and death for confirmed cases for observed data when number of events >5 and from a logistic regression prediction otherwise eTable 12. ln odds (standard errors) used in simulation modelling eTable 13. Parametrisation of SARS-CoV-2 asymptomatic cases eTable 14. Disability rate distribution and duration by SARS-CoV-2 category eTable 15. Resource use assumptions by SARS-CoV-2 category eTable 16. Quantity inputs for Hospital (inpatient and ICU) length of stay eTable 17. Unit costs for healthcare resource use (2019 AUD) eTable 18. Cost per patient category eAppendix 5. Road Traffic Crash eFigure 3. Apple mobility index for Victoria: Driver routing requests (100 = January Baseline) eFigure 4. Victorian Traffic Fatalities by Month (Jan-Sep) eTable 19. Association of Apple Driving Mobility Indices on Road Traffic Deaths eTable 20. Apple mobility driving index for stages 2, 3, and 4 of COVID-19 policy restrictions eTable 21. Estimated changes in RTC rates for stages 1b and 1 eReferences
