# Supplementary material for: Association of Simulated COVID-19 Policy Responses for Social Restrictions and Lockdowns With Health-Adjusted Life-Years and Costs in Victoria, Australia
Source: JAMA Health Forum. 2021 Jul 30;2(7):e211749. doi: 10.1001/jamahealthforum.2021.1749 (PMC8796885; doi:10.1001/jamahealthforum.2021.1749)
Supplement: Supplement. — eTable 1. Physical distancing and other measures by ‘stage’ as used in Victoria, Australia, for control of COVID-19 eTable 2. Conceptualisation and specification of the triggers to shift between stages by policy scenario eTable 3. Parameter estimates and ‘agent’ characteristics most relevant to current paper used in the agent-based model (for full details see source code and ODD protocol in footnote to this table) eTable 4. Key input parameters by level of policy stringency in the ABM eTable 5. Input parameters to PMSLT (excluding those from ABM) and GDP costs (inputs in italics only used in sensitivity analyses) eTable 6. Outputs from ABM, and estimated GDP loss, for ‘best’ scenario: 12 months intervention or ABM time horizon (i.e., assumed vaccination available in 12 months); 1% probability per day of incursion of infected person into Victoria in elimination strategies eTable 7. Estimate incremental health loss (HALYs) loss compared to BAU (i.e., no SARS-CoV-2 pandemic) and additional health expenditure (3% discount rate) eTable 8. Sensitivity analyses incremental health loss (HALYs) compared to BAU (i.e., no SARS-CoV-2 pandemic) and additional health expenditure and GDP loss (3% discount rate; in US$ millions), using the median infection rate across 100 simulations (% in parentheses are change relative to baseline) eFigure 1. Stages and triggers for tight suppression eAppendix 1. Estimates of GDP loss by stage, from Australian and Victorian Treasuries eTable 9. Estimates of per week GDP loss by stage, relative to no restrictions. Scope included GDP losses caused by Victorian restrictions, but borne beyond Victoria eAppendix 2. Net Monetary Benefit eAppendix 3. Average citizen annual health expenditure eTable 10. Per-capita annual health expenditure in Australia, by sex by age (2019 US$) eAppendix 4. SARS-CoV-2 parameters eTable 11. Numbers of confirmed SARS-CoV-2 cases, hospitalisations, ICU admission, and deaths in Victoria, Australia eFigure 2. Ln odds of hospi [file jamahealthforum-e211749-s001.pdf]

Supplemental Online Content

Blakely T, Thompson J, Bablani L, et al. Association of simulated COVID-19 policy responses for social restrictions and lockdowns with health-adjusted life-years and costs in Victoria, Australia. *JAMA Health Forum*. 2021;2(7):e211749. doi:10.1001/jamahealthforum.2021.1749

eTable 1. Physical distancing and other measures by 'stage' as used in Victoria, Australia, for control of COVID-19

eTable 2. Conceptualisation and specification of the triggers to shift between stages by policy scenario

eTable 3. Parameter estimates and 'agent' characteristics most relevant to current paper used in the agent-based model (for full details see source code and ODD protocol in footnote to this table)

eTable 4. Key input parameters by level of policy stringency in the ABM

eTable 5. Input parameters to PMSLT (excluding those from ABM) and GDP costs (inputs in *italics* only used in sensitivity analyses)

eTable 6. Outputs from ABM, and estimated GDP loss, for 'best' scenario: 12 months intervention or ABM time horizon (i.e., assumed vaccination available in 12 months); 1% probability per day of incursion of infected person into Victoria in elimination strategies

eTable 7. Estimate incremental health loss (HALYs) loss compared to BAU (i.e., no SARS-CoV-2 pandemic) and additional health expenditure (3% discount rate)

eTable 8. Sensitivity analyses incremental health loss (HALYs) compared to BAU (i.e., no SARS-CoV-2 pandemic) and additional health expenditure and GDP loss (3% discount rate; in US$ millions), using the median infection rate across 100 simulations (% in parentheses are change relative to baseline)

eFigure 1. Stages and triggers for tight suppression

eAppendix 1. Estimates of GDP loss by stage, from Australian and Victorian Treasuries

eTable 9. Estimates of per week GDP loss by stage, relative to no restrictions. Scope included GDP losses caused by Victorian restrictions, but borne beyond Victoria

eAppendix 2. Net Monetary Benefit

eAppendix 3. Average citizen annual health expenditure

eTable 10. Per-capita annual health expenditure in Australia, by sex by age (2019 US$)

eAppendix 4. SARS-CoV-2 parameters

© 2021 Blakely T et al. *JAMA Health Forum.*

eTable 11. Numbers of confirmed SARS-CoV-2 cases, hospitalisations, ICU admission, and deaths in Victoria, Australia

eFigure 2. Ln odds of hospitalisation, ICU admission, and death for confirmed cases for observed data when number of events >5 and from a logistic regression prediction otherwise

eTable 12. ln odds (standard errors) used in simulation modelling

eTable 13. Parametrisation of SARS-CoV-2 asymptomatic cases

eTable 14. Disability rate distribution and duration by SARS-CoV-2 category

eTable 15. Resource use assumptions by SARS-CoV-2 category

eTable 16. Quantity inputs for Hospital (inpatient and ICU) length of stay

eTable 17. Unit costs for healthcare resource use (2019 AUD)

eTable 18. Cost per patient category

eAppendix 5. Road Traffic Crash

eFigure 3. Apple mobility index for Victoria: Driver routing requests (100 = January Baseline)

eFigure 4. Victorian Traffic Fatalities by Month (Jan-Sep)

eTable 19. Association of Apple Driving Mobility Indices on Road Traffic Deaths

eTable 20. Apple mobility driving index for stages 2, 3, and 4 of COVID-19 policy restrictions

eTable 21. Estimated changes in RTC rates for stages 1b and 1

eReferences

This supplemental material has been provided by the authors to give readers additional information about their work.

© 2021 Blakely T et al. *JAMA Health Forum.*

Supplementary Tables, Figures, and Appendices

eTable 1: Physical distancing and other measures by 'stage' as used in Victoria, Australia, for control of COVID-19

Domain	Condition	Stage 1	Stage 1b	Stage 2	Stage 3	Stage 4
Stay at home	Number of reasons to leave home†	-	-	5	4	4
	Limit on range of movement	-	-	-	5 km	5km
	Time away from home	-	-	-	2 hours	1 hour
	Limit on the number of times you can go out per day	-	-	-	-	1
	Curfew (8:00pm – 5:00am)	No	No	No	No	Yes
	Work from home	Return to work	If you can	If you can	If you can	Stay at home, unless defined essential worker
Home visitors (non-household members)	Maximum number (N) of visitors	100	20	5	0	0
Outdoor gatherings	Maximum N of persons (including for physical activity / exercise)	100	20	10	5	2
Industries, education, hospitality facilities (% closed unless otherwise stated)	Major construction sites	0%	0%	0%	0%	75%
	Small scale construction, e.g., residential (max number of people on site)	-	-	-	-	5
	Meat industry	0%	0%	0%	0%	33%
	Poultry industry	0%	0%	0%	0%	20%
	Seafood industry	0%	0%	0%	0%	33%
	Manufacturing	0%	0%	0%	Only to supply essential services	Only to supply essential services
	Warehousing & distribution centres	0%	0%	0%	0%	0%
	Technical and further education, & University studies	Opening gradually	Opening gradually	Opening gradually	Mostly remote learning	Only remote learning
	Schools	Open	Open	Open	Closed (except to vulnerable children and children of permitted workers	Closed (except to vulnerable children and children of permitted workers

© 2021 Blakely T et al. *JAMA Health Forum.*

Domain	Condition	Stage 1	Stage 1b	Stage 2	Stage 3	Stage 4
	Childcare & pre-school care	Open	Open	Open	Open	Closed (except to vulnerable children and children of permitted workers
	Hardware stores	0%	0%	0%	0%	Closed – exception to tradespeople
	Department stores	0%	0%	0%	0%	100%
	Hairdressers & barbershops	0%	0%	0%	0%	100%
	Beauty parlours & massage therapy	0%	0%	100%	100%	100%
	Real estate auction – max N	100	20	15	0	0
	Accommodation services – Closed	No	No	No	Yes	Yes
	Café & restaurants – m^2 per person	4	4	-	-	-
	Café & restaurant – max N	100	20	0	0	0
	Café & restaurant – closed	No	No	Yes	Yes	Yes
	Food courts – Closed	No	Yes	Yes	Yes	Yes
	Pubs, clubs, casinos & nightclubs	No	Yes	Yes	Yes	Yes
	Cinemas & entertainment services	100	20	0	0	0
Places of worship	Closed	No	No	No	Yes	Yes
	Maximum N allowed	100	22	12	0	0
	M^2 per person	-	4	4	-	-
	Weddings – maximum N allowed	100	23	13	5	0
	Indoor funerals – max N allowed	100	52	22	12	12
	Outdoor funerals – max N allowed	100	52	32	12	12
Face covering ‡		No	In public transport and indoor environment if not with household members	In public transport and indoor environment if not with household members	Mandatory out of home, unless doing vigorous physical activity	Mandatory out of home, unless doing vigorous physical activity
Sporting activities	Indoor sports – m^2 per person	-	4	-	-	-
	Indoor sporting centres – max N	100	20	0	0	0
	Gym – max N	100	20	0	0	0

© 2021 Blakely T et al. *JAMA Health Forum.*

Domain	Condition	Stage 1	Stage 1b	Stage 2	Stage 3	Stage 4
	Play centres - % closed	0%	0%	100%	100%	100%
	Playgrounds - % closed	0%	0%	100%	100%	100%
	Recreation activities (fishing, golf, boating, tennis, surfing, drive range shooting) - % closed	0%	0%	0%	Allowed with one person	100%
Aged care restrictions	Max N visitors at one time	2	2	2	0	0
	Max N of visits per day per resident	2	2	2	0	0
	Max total duration of visits (in hours)	2	2	2	0	0
	Face masks required of visitors	If asked	If asked	If asked	Mandatory	Mandatory
	Workers working at multiple facilities	Allowed	Allowed	Allowed	Not allowed	Not allowed
	Facemask required of workers	No	Mandatory	Mandatory	Mandatory	Mandatory

† At Stage 3 and 4, four reasons are: for essential work; for shopping (e.g., groceries); to give or receive care; for physical activity. At Stage 2, seeing friends and family added.

‡ This face covering usage is how it played out with escalating application of stages in Victoria. Going forward, it is likely that face coverings will be mandatory at all Stages – perhaps only on public transport and in busy indoor public spaces in stages 1, 1b and 2.

© 2021 Blakely T et al. *JAMA Health Forum.*

eTable 2: Conceptualisation and specification of the triggers to shift between stages by policy scenario

1. Aggressive elimination †	2. Moderate elimination †	3. Tight suppression (aiming for 1 to 5 cases per million population per day)‡	4. Loose suppression (aiming for 5 to 25 cases per million population per day)‡
**First, check for tightening (below rules applied on each day, regardless of Stage currently in)**			
If in Stage 3 or lower, and average >7.5 cases per day for last 7 days, go to Stage 4<br>Else:<br>If in Stage 2 or lower, and average > 1.5 cases per day for last 7 days, go to Stage 3<br>Else:<br>If in Stage 1b or 1, and two or more cases in last 14 days, go to Stage 2<br>Else:<br>If in Stage 1, and any single case, go to Stage 1b<br>Else:<br>Stay in same stage.	If in Stage 3 or lower, and average >30 cases per day for last 7 days, go to-Stage 4<br>Else:<br>If in Stage 2 or lower, and average > 6 cases per day for last 7 days, go to Stage 3<br>Else:<br>If in Stage 1b or 1, and five or more cases in last 14 days, go to Stage 2<br>Else:<br>If in Stage 1, and two or more cases in last 14 days, go to Stage 1b<br>Else:<br>Stay in same stage	If >20 per million in last 7 days, go to Stage 4.<br>Else:<br>If > 10 per million in last 7 days, go to Stage 3.<br>Else:<br>If average of >5 per million in last 7 days, <u>and</u> > 6 days since last tightening, tighten 1 stage<br>Else:<br>Stay in same stage	If >100 per million per day in last 7 days, go to Stage 4.<br>Else:<br>If > 50 per million per day in last 7 days, go to Stage 3.<br>Else:<br>If average of >25 per million per day in last 7 days, <u>and</u> > 6 days since last tightening, tighten 1 stage<br>Else:<br>Stay in same stage
**Second, check for loosening**			
If in Stage 4, and average <5 cases per day for last 7 days, and > 20 days since last loosening, go to Stage 3<br>Else:<br>If in Stage 3, and average < 1 case per day for last 7 days, and > 20 days since last loosening, go to Stage 2<br>Else:<br>If in Stage 2, and zero cases for last 7 days, and > 20 days since last loosening, go to Stage 1b<br>Else:<br>If in Stage 1b, and zero cases for last 28 days and > 20 days since last loosening, go to Stage 1<br>Else:<br>Stay in same stage	If in Stage 4, and average <20 cases per day for last 7 days, and > 20 days since last loosening, go to Stage 3<br>Else:<br>If in Stage 3, and average < 5 cases per day for last 7 days, and > 20 days since last loosening, go to Stage 2<br>Else:<br>If in Stage 2, and <1 case per day for last 7 days, and > 20 days since last loosening, go to Stage 1b<br>Else:<br>If in Stage 1b, and zero cases for last 7 days and > 20 days since last loosening, go to Stage 1<br>Else:<br>Stay in same stage	If average of <2.5 per million in last 7 days, and > 20 days since last loosening, loosen 1 stage<br>Else:<br>Stay in same stage	If average of <12.5 per million per day in last 7 days, and > 20 days since last loosening, loosen 1 stage<br>Else:<br>Stay in same stage

‡ 1 case per million per day equates to an expected 6.4 cases per day in Victoria. A flow diagram of tight suppression is given in Supplementary Figure 1.

© 2021 Blakely T et al. *JAMA Health Forum.*

eTable 3: Parameter estimates and 'agent' characteristics most relevant to current paper used in the agent-based model (for full details see source code and ODD protocol in footnote to this table)

Key Parameters	Parameter Estimates (Policies 1, 2, 3, 4)
**Physical distancing** (% of people limiting movement and maintaining a distance of 1.5m in publi**c**, normal distribution)**¥** [1]	m = 85%, sd = 3%
**Physical distancing - time** (% of time that people successfully maintain a distance of 1.5m, normal distribution)**¥**[1]	m = 85%, sd = 3%
**Mean incubation period** (days, log-normal)[2]	m = 5.1, sd = 1.5
**Mean illness period** (days, log-normal)[3]	m = 20.8, sd = 2
**Mean adherence with isolation of infected cases** (beta distribution) ¥	m = 93.3%<br>(beta 28, 2; median = 94.3%, SD = 4.5%) †
**Number of days after initial infection that new cases are reported**¥*	6*
**Date of case simulation initialisation** (Day 0)	September 1st, 2020
**Asymptomatic cases** (% of cases, normal distribution) [3,4]	m = 33%, sd = 3%
**Infectiousness of asymptomatic cases vs symptomatic cases (per contact,** normal distribution**)** [5]	m = 33%, sd = 6%
**Reduction in transmission risk per contact for people wearing facemasks** (beta distribution) **¥¥¥**	77%[6] (beta 24.3, 8.08)†
**Seeded cases**	An initial volume of 2400 active cases were seeded into the model on day 0. This was followed by 7 days of 80 cases per day.
**COVID-Safe Electronic App Uptake** (normal distribution)	m = 30%
**Agent Characteristics**	**Definition**
**Infection status**	Susceptible, Infected, recovered, deceased
**Time now**	The number of days (integer) since an infected person first became infected with SARS-CoV-2
**Age-range**	The age-bracket (categorical) of the person, set to census data deciles from 0 to 100. Used in this simulation to capture differences in exposure risk through school closures and workforce status.
**Risk of death**	The overall risk of death (float) *for* each person based on their age-profile. Purely used in this simulation to remove the agents dying during the 100-day simulation time.
**Location**	Agents interact in over a 2-dimensional plane with their location recorded at each time-step via an x/y coordinate system.

© 2021 Blakely T et al. *JAMA Health Forum.*

**Span**	The distance the person moves around the environment away from their home location – longer distances result in higher likelihood of close contact with novel other people (agents) in the model.
**Heading / Distance**	The direction and extent of travel of the person at the current time-step. The heading and speed variables combine to create local communities and control interaction between and across communities. At higher lockdown stringency levels, agents are restricted to movement in areas closer to their home location.
**Contacts**	A count (integer) of contacts the person (agent) had interacted with in the past day as they moved within the model's environment. This is used in estimation of contacts with transmission potential each time-step and calculation of individual reproduction numbers at the end of infectious periods.

Code for ABM at: https://github.com/JTHooker/COVIDModel (last accessed 23 August 2020).

ODD protocol at: https://github.com/JTHooker/COVIDModel/blob/master/ODD%20Protocol%20Aus%20NZ%20COVID19%20model.pdf (last accessed 23 August 2020).

¥ Assumed parameter based on expert opinion in conjunctions with available public data sources such as Google COVID-19 mobility reports.

¥¥ 10% of the population potentially transmit infections widely through occasional travel to random locations.

¥¥¥ The source paper reports an adjusted odds ratio of 0.15 for a systematic review of observational studies. Given possible residual confounding, and to be conservative, we used 80% rather than 85%.

*This reports all cases known to the model user on day 6 of their infection. In alternative modes, code also allows for under-reporting under extreme pressure on the track and trace system (e.g., in unmitigated scenarios).

₤ % mask wearing is fixed part of scenario, therefore no uncertainty.

© 2021 Blakely T et al. *JAMA Health Forum.*

eTable 4: Key input parameters by level of policy stringency in the ABM

Condition	Stage 0	Stage 1	Stage 2	Stage 3	Stage 4
% of working age adults classified as essential workers (with no restrictions on movements during work hours) *[no uncertainty, as part of scenario definition]*	100%	75%	50%	25%	20%
Restrictions on non-essential workers, and essential workers when not working:					
% of people with restricted movement	0%	25%	65%	85%	90%
% restriction in movement among the above restricted people	0%	25%	65%	85%	90%
Complacency: Minimal value that restrictions above reduce to as a result of fatigue €	0%	15%	52%	68%	81%
Radius of movement in spatial units for non-essential workers, and essential workers when not working †	30	30	15	10	5
Quarantine compliance ₵ – *beta distribution*	93%	93%	93%	93%	93%
Super-spreader potential (generated by allowing a percentage of agents to randomly move to a new location at any time-step)	10%	10%	10%	5%	2%
Limitations on gathering restrictions over time – opportunities per week to gather in locations (average of once per week) and the potential area in spatial units within which people may be drawn from. The larger the area, the greater the number of potential contacts.	78.5 area units	50.2 units	28.3 units	12.6 units	3.1 units
School closures, all children < 18 years ‡ *[no uncertainty, as part of scenario definition]*	0%	0%	0%	90%	100%
Mask utilisation outside of home in busy indoor environments, selected outside environments (e.g., sporting venues) and public transport where physical distancing is not possible.	50%	90%	90%	90%	90%
Mask effectiveness in reducing transmission ₤ *[Beta distribution 24.3, 8.08]*	75%	75%	75%	75%	75%
% of population with COVID-Safe App on their phone	30%	30%	30%	30%	30%
% reduction in contact tracing time due to COVID-Safe App, when both people have the App	50%	50%	50%	50%	50%

† The range of movement is in a two-dimensional plane, meaning the relative difference in number of destinations is a function of the quadratic, e.g., for Stage 5 c.f. Stage 1, 25 to 4 relative difference.

‡ For this paper, all children <18 years treated the same (but can be stratified in extensions to modelling)

€ At each time-step, both the proportion of people who complied with social distancing measures and the proportion of time they complied declined by 1 unit to the baseline level set at each stage.

₤ A recent systematic review found a pooled OR for reduced transmission of 0.85 (or 85%), in mostly clinical studies and some community studies.[7] This probably overestimate effectiveness in real-life. We therefore specified a beta distribution 24.3 and 8.08, giving mean 0.75, SD 0.075, 95% uncertainty interval 0.590 to 0.881.

© 2021 Blakely T et al. *JAMA Health Forum.*

eTable 5: Input parameters to PMSLT (excluding those from ABM) and GDP costs (inputs in *italics* only used in sensitivity analyses)

Input	Specification	Uncertainty	Comment and source
Population counts	Estimated usually resident population 2020	Nil	UN World Population Prospects for Jul 2020 †
All-cause mortality rates	Single year of age mortality rates, generated from GBD five-year age group rates using interpolation on log scale.	Log normal approximation to GBD published 2.5th and 97.5th percentiles. ‡	IHME GHDx
All-cause morbidity rates	Single year of age prevalent years of life with disability (YLD) proportions	Log normal approximation to GBD published 2.5th and 97.5th percentiles. ‡	IHME GHDx
Cause-specific mortality rates (road traffic crash)	*GBD five-year age group mortality rates.*	*(Nil – only used in sensitivity analyses as expected values)*	*IHME GHDx*
Cause-specific morbidity rates (road traffic crash non-fatal injuries, depression, anxiety)	*Single year of age prevalent years of life with disability (YLD) proportions for these for conditions.*	*(Nil – only used in sensitivity analyses as expected values)*	*IHME GHDx*
Forecast annual percentage change (APC) in all-cause mortality rates	APC by sex by five-years age-groups for GBD mortality rates 1980-2017, used to forecast mortality rates to 2035 – then no change.	Nil	IHME GHDx
Total health system expenditure per person by sex and age	Average per-capita health expenditure by sex and age group.	5% SD log-normal distribution. ‡	Australia: AIHW [8]
Policy strategy (i.e., intervention) inputs			
SARS-CoV-2 mortality	Varies with SARS-CoV-2 infection rates and specified in detail in Appendix 3.	See Appendix 3. ‡	See Appendix 3.
SARS-CoV-2 morbidity	Symptomatic, not admitted = 0.084	95% UI 0.059 – 0.110	See Appendix 3.
	Admitted to hospital, no ICU = 0.096	95% UI 0.064 – 0.128	
	Admitted to ICU = 0.283	95% UI 0.208 – 0.359	
SARS-CoV-2 health expenditure	Symptomatic, not admitted = US$57.68	+/- 20% SD log normal distribution	See Appendix 3.
	Admitted to hospital, no ICU = US$14,324		
	Admitted to ICU =US$44,641		
GDP impacts	Varies by stage:<br>- US$0.4 billion/ week Stage 1<br>- US$0.6 billion/ week Stage 1b	+/- 20% SD log normal distribution. Correlated 1.0 across timesteps.	See Appendix 1.

© 2021 Blakely T et al. *JAMA Health Forum.*

Input	Specification	Uncertainty	Comment and source
	- US$0.725 billion/ week Stage 2<br>- US$1.275 billion/ week Stage 3<br>- US$2.61 billion/ week Stage 4		
Road traffic crash (RTC) rates	*The correspondence to changes in Apple mobility data to changes in Victoria RTC rates was used to generate the following estimates: -23.6%, -21.9%, -8.74%, -1.53%, -1.45% reductions for stages 4, 3, 2, 1b and 1, respectively.*	*(Nil – only used in sensitivity analyses as expected values)*	*See Appendix 4 for details.*
Depression and anxiety	*We assumed the following percentage increases in both depression and anxiety: 10%, 8%, 6%, 4% and 2% for stages 4, 3, 2, 1b and 1, respectively.*	*(Nil – only used in sensitivity analyses as expected values)*	*See main paper for details.*

Footnotes:

Values for Australia were applied to Victoria (except population being scaled).

Abbreviations: AIHW = Australian Institute of Health and Welfare; GHDx = Global Health Data Exchange; IHME = Institute of Health Metrics and Evaluation; (http://ghdx.healthdata.org/);

† https://population.un.org/wpp/Download/Standard/Population/

‡ Each set of variables, for instance, variations in infection fatality rates by age and sex, have a 0.5 correlation within each iteration. All variables assumed to be correlated perfectly (1.0) across time-steps within each iteration.

© 2021 Blakely T et al. *JAMA Health Forum.*

eTable 6: Outputs from ABM, and estimated GDP loss, for 'best' scenario: 12 months intervention or ABM time horizon (i.e., assumed vaccination available in 12 months); 1% probability per day of incursion of infected person into Victoria in elimination strategies

	**Strategy**	**a) Aggressive elimination**	**b) Moderate elimination**	**c) Tight Suppression**	**d) Loose suppression**
SARS-CoV-2 cases (90% UI)	In first month	1,330 (1,040 – 1,580)	1,350 (1,110 – 1,600)	1,260 (1,020 – 1,500)	1,260 (1,090 – 1,540)
	Month 2-6 incl	173 (49 – 557)	229 (50 – 642)	4,570 (943 – 13,300)	23,200 (119 – 37,700)
	Month 7-12 incl	18 (0 – 54)	34 (0 – 104)	5,640 (1,070 – 21,600)	35,100 (6,160 – 48,900
	Month 13-18 incl	13 (0 – 55)	31 (0 – 93)	4,160 (17 – 19,600)	9,940 (128 – 71,800)
	Up to 12 months	1,530 (1,150 – 2,030)	1,650 (1,240 – 2,110)	11,700 (6,520 – 32,200)	55,900 (28,700 – 82,300)
	Up to 18 months for scenario	1,540 (1,150 – 2,050)	1,680 (1,280 – 2,180)	17,900 (6,970 – 41,700)	77,100 (35,900 – 129,000)
Percentage (90% UI) of days in each level	1	21.7% (2.1% - 53.9%)	31.7% (6.6% - 64.4%)	15.1% (5.3% - 39.7%)	16.3% (5.0% - 40.9%)
	1b	16.3% (8.1% - 25.6%)	11.9% (5.8% - 22.8%)	13.9% (10.3% - 18.7%)	14.4% (11.2% - 32.6%)
	2	12.1% (5.8% - 19.7%)	13.8% (5.8% - 21.9%)	12.8% (11.7% - 21.4%)	11.7% (11.5% - 19.7%)
	3	18.3% (8.1% - 27.2%)	16.4% (8.1% - 26.7%)	15.7% (13.3% - 25.0%)	14.7% (12.2% - 20.8%)
	4	30.3% (18.0% - 41.2%)	27.5% (15.8% - 33.7%)	34.3% (20.0% - 49.5%)	36.1% (22.4% - 46.2%)
Estimated GDP loss (90% UI)	In first month	7.9 (5.8 – 10.8)	7.9 (5.8 – 10.8)	7.9 (5.8 – 10.8)	7.9 (5.8 – 10.8)
	Month 2-6 incl	22.6 (15.8 – 36.7)	21.1 (14.0 – 33.6)	19.3 (12.1 – 26.6)	17.5 (11.1 – 25.2)
	Month 7-12 incl	15.6 (7.4 – 25.6)	13.3 (7.0 – 22.8)	23.2 (11.4 – 37.7)	25.3 (11.6 – 34.8)
	Month 13-18 incl	15.4 (7.9 – 25.8)	14.6 (7.7 – 22.5)	21.3 (7.7 – 32.4)	17.4 (7.4 – 33.1)
	Up to 12 months	46.5 (31.9 – 67.6)	41.7 (29.0 – 63.6)	50.9 (35.2 – 70.2)	50.2 (35.1 – 72.5)
	Up to 18 months for scenario	61.3 (45.5 – 89.6)	56.7 (39.9 – 79.2)	71.7 (46.9 – 93.3)	67.5 (44.3 – 97.4)
	Up to 12 months as % of 2019 Victorian State GDP †	15.1% (10.4% - 21.9%)	13.5% (9.4% - 20.6%)	16.5% (11.4% - 22.8%)	16.3% (11.4% - 23.5%)

† Victorian Gross State Product (GSP) was Aus $454.59 billion in 2019, or US $308.20 billion (using OECD purchasing power parity).

There were large economic stimulus packages in Australian, most notably an Aus $507 billion Federal Government stimulus (KPMG, last updated 18 Nov 2020, accessed 2 May 2021; https://home.kpmg/xx/en/home/insights/2020/04/australia-government-and-institution-measures-in-response-to-covid.html) – of which perhaps a quarter to a third was directed to Victoria. Whilst not all of this would have flowed through in the year post-ceding the Victorian second wave, it does explain why the pre-stimulus estimates in this table show a percentage loss in GDP of greater magnitude than that actually observed (e.g. a 1.1% fall for calendar year 2020 compared to 2019 for all of Australia; https://www.abs.gov.au/statistics/economy/national-accounts/australian-national-accounts-national-income-expenditure-and-product/latest-release, accessed 2 May 2021). That is, our estimates are do not include the offsetting stimulus impacts on GSP and GDP.

© 2021 Blakely T et al. *JAMA Health Forum.*

eTable 7: Estimate incremental health loss (HALYs) loss compared to BAU (i.e., no SARS-CoV-2 pandemic) and additional health expenditure (3% discount rate)

	Strategy	a) Aggressive elimination	b) Moderate elimination	c) Tight Suppression	d) Loose suppression
SARS-CoV-2 deaths	1st yr.	58 (46, 82)	64 (46, 88)	483 (245, 1,358)	2,249 (1,022, 3,555)
HALYs loss compared to non-COVID-19 BAU † (90% UI)	1st yr.	38 (29, 50)	41 (30, 54)	156 (98, 364)	672 (337, 954)
	1st & 2nd yr.	71 (54, 94)	76 (56, 101)	436 (246, 1,120)	1,990 (1,020, 2,960)
	Full 20 years	272 (204, 363)	289 (215, 386)	1,950 (1,100, 6,100)	9,500 (4,730, 15,500)
	Rest of lifetime	286 (219, 389)	314 (228, 413)	2,263 (1,180, 6,550)	11,000 (5,030, 16,700)
Net health expenditure increase compared to non-COVID-19 BAU † (in $US millions; 90% UI)	1st yr.	2.71 (1.49, 3.82)	2.88 (1.69, 4.61)	24.6 (10.4, 75.3)	117 (50, 214)
	1st & 2nd yr.	2.00 (0.994, 2.99)	2.10 (1.12, 3.61)	19.2 (7.19, 59.1)	89.0 (33.1, 169)
	Full 20 years	-2.54 (-3.95, -1.68)	-2.77 (-3.88, -1.79)	-18.5 (-51.5, -8.23)	-90.2 (-153, -30.6)
	Rest of lifetime	-2.99 (-4.45, -2.00)	-3.19 (-4.51, -2.22)	-22.4 (-60.1, -9.92)	-107 (-175, -41.7)

† HALYS for the Victorian population (3% discount rate) over the remainder of their lifetime in BAU were 127 million, and health expenditure (also 3% discount rate) was $US 1021 billion. Thus, by way of comparison, the HALY loss as percentage of BAU HALYs was 0.0002% from aggressive and moderate elimination, 0.0015% from tight suppression was 0.0075% from loose suppression.

.

© 2021 Blakely T et al. *JAMA Health Forum.*

eTable 8: Sensitivity analyses incremental health loss (HALYs) compared to BAU (i.e., no SARS-CoV-2 pandemic) and additional health expenditure and GDP loss (3% discount rate; in US$ millions), using the median infection rate across 100 simulations (% in parentheses are change relative to baseline)

Sensitivity analysis	Measure (lifetime)	a) Aggressive elimination	b) Moderate elimination	c) Tight Suppression	d) Loose suppression
Baseline †	HALY loss	289	308	2,080	12,700
	Net health Costs	-3.13	-3.31	-21.8	-131
	Net health + GDP costs	46,381	41,658	50,808	50,235
	Optimal from health perspective	*> $10,000*			*<$10,000*
	Optimal from partial societal perspective		*$0 to $500,000*		
Discount rate					
0%	HALY loss	367 (27%)	391 (27%)	2,670 (28%)	16,500 (30%)
	Net health Costs	-4.94 (58%)	-5.24 (58%)	-35.2 (61%)	-215 (64%)
	Net health + GDP costs	46,378 (0%)	41,655 (0%)	50,792 (0%)	50,171 (0%)
	Optimal from health perspective	*> $15,000*			*<$15,000*
	Optimal from partial societal perspective		*$0 to $500,000*		
6%	HALY loss	239 (-17%)	253 (-18%)	1,690 (-19%)	10,300 (-19%)
	Net health Costs	-1.97 (-37%)	-2.07 (-37%)	--13.3 (-39%)	-78.3 (-40%)
	Net health + GDP costs	46,383 (0%)	41,660 (0%)	50,817 (0%)	50,275 (0%)
	Optimal from health perspective	*> $10,000*			*<$10,000*
	Optimal from partial societal perspective		*$0 to $500,000*		
Varying timeline to vaccine (from 12 months in base model)					
6 months	HALY loss	283 (-2%)	300 (-3%)	1,050 (-50%)	4,340 (-67%)
	Net health Costs	-3.07 (-2%)	-3.25 (-2%)	-11.2 (-49%)	-45.8 (-65%)
	Net health + GDP costs	30,419 (-34%)	28,604 (-31%)	26,978 (-47%)	25,033 (-50%)
	Optimal from health perspective	*> $10,000*			*<$10,000*
	Optimal from partial societal perspective				*$0 to $500,000*
18 months	HALY loss	291 (1%)	312 (1%)	3,000 (44%)	15,000 (18%)
	Net health Costs	-3.15 (1%)	-3.36 (2%)	-30.9 (42%)	-154 (18%)
	Net health + GDP costs	61,233 (32%)	56,723 (36%)	71,328 (40%)	67,716 (35%)
	Optimal from health perspective	*> $10,000*			*<$10,000*
	Optimal from partial societal perspective		*$0 to $500,000*		
Including additional diseases					

© 2021 Blakely T et al. *JAMA Health Forum.*

Sensitivity analysis	Measure (lifetime)	a) Aggressive elimination	b) Moderate elimination	c) Tight Suppression	d) Loose suppression
Road traffic crash (RTC)	HALY loss	-661 (-329%)	-684 (-322%)	914 (-56%)	11,900 (-6%)
	Net health Costs	4.99 (-259%)	5.18 (-256%)	-11.8 (-46%)	-123 (-6%)
	Net health + GDP costs	46,391 (0%)	41,668 (0%)	50,820 (0%)	50,243 (0%)
	Optimal from health perspective		> $10,000		<$10,000
	Optimal from partial societal perspective		$0 to $500,000		
Depression and anxiety	HALY loss	738 (155%)	762 (147%)	2,620 (26%)	13,200 (4%)
	Net health Costs	-3.13 (0%)	-3.31 (0%)	-21.8 (0%)	-131 (0%)
	Net health + GDP costs	46,381 (0%)	41,658 (0%)	50,808 (0%)	50,235 (0%)
	Optimal from health perspective	> $10,000			<$10,000
	Optimal from partial societal perspective		$0 to $500,000		
RTC, depression & anxiety	HALY loss	-212 (-173%)	-229 (-174%)	1,460 (-30%)	12,300 (-3%)
	Net health Costs	4.99 (-259%)	5.18 (-256%)	-11.8 (-46%)	-123 (-6%)
	Net health + GDP costs	46,391 (0%)	41,668 (0%)	50,820 (0%)	50,243 (0%)
	Optimal from health perspective		> $10,000		<$10,000
	Optimal from partial societal perspective		$0 to $500,000		
Contact tracing (effectiveness improves inversely with the log of daily cases)					
	HALY loss	317 (10%)	332 (8%)	3,470 (67%)	14,300 (13%)
	Net health Costs	-3.43 (10%)	-3.59 (8%)	-35.8 (64%)	-148 (13%)
	Net health + GDP costs	47,766 (3%)	44,356 (6%)	53,845 (6%)	54,548 (9%)
	Optimal from health perspective	> $10,000			<$10,000
	Optimal from partial societal perspective		$0 to $500,000		

© 2021 Blakely T et al. *JAMA Health Forum.*

eFigure 1: Stages and triggers for tight suppression

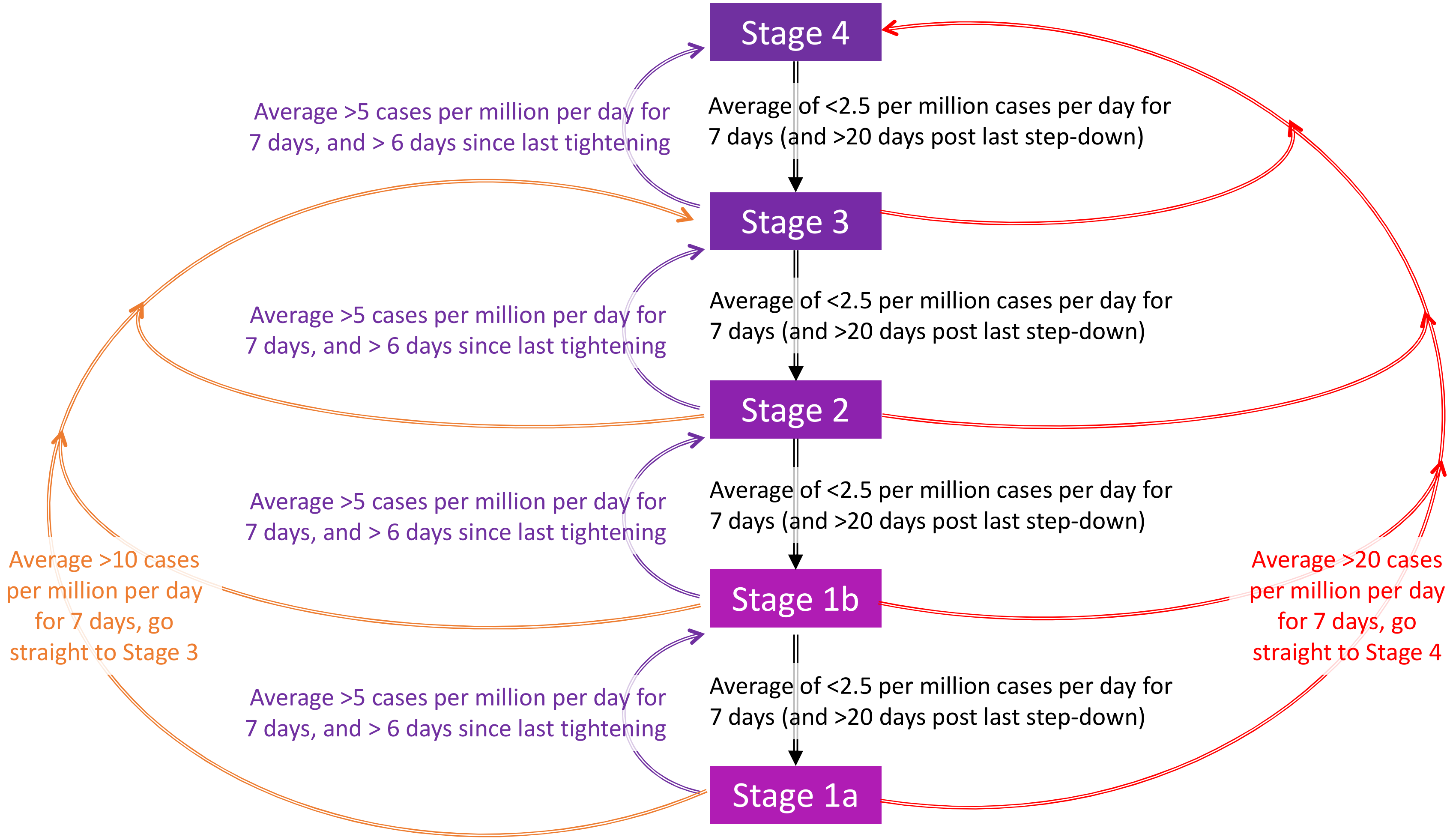

© 2021 Blakely T et al. *JAMA Health Forum.*

eAppendix 1: Estimates of GDP loss by stage, from Australian and Victorian Treasuries

State and Commonwealth treasuries in Australia provided estimates of the impact of COVID-19. We prioritised the use of such government estimates since these are usually bolstered by a wide array of near real-time indicators, for instance, income and sales tax collection data.

The Victorian Treasury in its July 2020 update, before the announcement of Stage 4 restrictions, estimated that a combination of approximately six weeks of Stage 3 and six weeks of various Stage 2 lockdowns in the April to June and July to September quarters would reduce GDP by 11 per cent (or roughly 1 billion dollars a week compared to expected GDP had there been no COVID-19).[9]

For each stage, the broader Australian economy had a smaller impact from equivalent restrictions, in part due to a smaller reliance outside of the State of Victoria on hospitality and the resilience of iron ore prices. The Australian Department of Finance in its Mid-Year Economic and Financial Update estimated that Stage 3 restrictions between Mar 30 and mid-May had an estimated Australia-wide cost of AUD 4 billion per week, around, roughly 11 per cent of the weekly Australian GDP of AUD 36.3 billion dollars (~AUD1.89 trillion/52). It estimated the increment between Stage 3 and the 'unlocked' economy to be around AUD 2bn per week or around 5.5% of GDP. Finally, the prime minister of Australia announced on 6 Aug 2020 [10], that the cost of six weeks of Victorian Stage 3 restrictions in the Jul-Sep quarter would be around AUD 3.3 billion, and the cost of six weeks of Stage 4 restrictions, incremental to Stage 3, was AUD 7 to 9 billion. Approximately 80 per cent, or $6 billion to $7 billion, was expected to arise from businesses and activity in Victoria, while the remainder cost was borne by the rest of Australia due to spill-over effects.

Based on these announcements and using a scope of including GDP losses caused by Victoria even if borne beyond Victoria, we estimate the impact of the COVID-19 control strategies to be approximately as shown below in Supplementary Table 9.

© 2021 Blakely T et al. *JAMA Health Forum*.

eTable 9: Estimates of per week GDP loss by stage, relative to no restrictions. Scope included GDP losses caused by Victorian restrictions, but borne beyond Victoria

Stage	Total GDP costs for each stage per week, relative to no restrictions (i.e. pre-COVID; AUD b,)	Incremental GDP costs for each higher stage, per week (AUD bn)	Rationale
Stage 1	$0.5	--	2 bn/week cost of most relaxed Stage estimated for all of Australia [11]; Victoria ~ 0.25% of Australian economy.
Stage 1b	$0.535	$0.035	Includes incremental 150 million monthly turnover impact based on 70% turnover growth observed in NSW between May and August. [12]<br><br>(Minimal impact on take-away services. Minimal incremental impact on real estate auctions.)
Stage 2	$0.725	$0.19	Backed out from incremental cost of Stage 3 over Stage 2 by Australian Treasury; VIC treasury forecasts of combined Stage 2-Stage 3 impacts.[9]<br>$0.5x+0.5(x+0.55) =1$<br>$\rightarrow x + 0.275 = 1$<br>$\rightarrow x = 0.725$
Stage 3	$1.275	$0.55	Prime Minister announcement/Australian Treasury's calculations of an incremental (compared to Stage 2) 0.55 billion costs per week for around 6 weeks of Stage 3 restrictions in VIC. [10],
Stage 4	2.61 (2.45-2.78)	1.33 (1.16-1.5)	Incremental cost of Stage 4 compared to Stage 3. Incremental costs of 7-9 billion over six weeks. [10],

Note: A 20% SD was assumed for all these inputs, to draw values from in Monte Carlo estimates to simulate uncertainty around outputs from modelling.

© 2021 Blakely T et al. *JAMA Health Forum*.

eAppendix 2: Net Monetary Benefit

We estimated the monetary benefit (NMB)[1] approach for each of the 100 runs:

$$NMB_{ijk} = \left(HALYs_{ik} \times WTP_j\right) - Cost_{ik}$$

Where:

- i indexes the 100 iterations
- j indexes the WTP
- k indexes the four policy scenarios
- and Cost is the net health expenditure for the health system perspective analyses, and from the societal perspective adds GDP costs to health system costs.

Within each iteration i and WTP j, the policy scenario with the highest NMB is selected. Across all 100 iterations, each policy response k will have a probability of having the highest NMB, and the policy option with the highest probability is deemed 'optimal' at that WTP. Finally, these outputs can be shown as cost effectiveness acceptability curves.

© 2021 Blakely T et al. *JAMA Health Forum.*

eAppendix 3: Average citizen annual health expenditure

Consistent with recommended practice in cost effectiveness analyses [13,14], in the USA [15] and the Netherlands[16], we included 'unrelated disease costs' in the economic evaluation. This means that in addition to including the costs of SARS-CoV-2 cases per se (Appendix 3), knock-on changes in health system expenditure are also included. For SARS-CoV-2, this means that if someone dies due to SARS-CoV-2 infections, their reduced health expenditure in the future is included (leading to a potentially net negative expenditure depending on the balance of costs, age and discount rate). In a simulation model, this is easy to incorporate, by including an expenditure reward per cycle in the model for diseases not explicitly modelled elsewhere – which in the case of SARS-CoV-2 modelling, is simply the expected annual (or monthly) average health system expenditure.

Data were extracted from the Australian Institute of Health and Welfare (AIHW) report 'Disease expenditure in Australia, which separates the total expenditure by sex and age.[8]

The data are from the 2015-16 financial year, where the total health expenditure totalled $170.4 billion $AU (2016). The AIHW attributed $106.857 billion of this spending to age and sex related health spending (62.7% of total health expenditure), with data provided as total expenditure by age and sex subgroup. [17]

We extracted population demographics from the Australian Bureau of Statistics (ABS) 2016 population report,[17] and the total health expenditure for each subgroup was then divided by the corresponding population numbers for these subgroups. Thus the 2016 health expenditure is expressed as per capita expenditure, by age and sex.[18]

The AIHW estimates variable health expenditure at 94% of total health expenditure,[8] whilst New Zealand variable expenditure is estimated at 91% total expenditure.[19] We elected to assume variable expenditure was 90%, allowing for fixed costs in running services Noting the above 62.7% of total health expenditure captured by AIHW estimates, we therefore multiplied all age by sex empirical estimates by a factor of 90/62.7 to generate the estimated predicted Australian variable health expenditure per capita, by age and sex.

Next, we inflation adjusted these expenditures from 2016 $AU to 2019 $AU using Australian CPI adjustment factors (OECD rates; [18] https://data.oecd.org/price/inflation-cpi.htm). Finally, we converted to 2019 USD using the AUD-USD 2019 purchasing power from the OECD.

© 2021 Blakely T et al. *JAMA Health Forum*.

eTable 10: Per-capita annual health expenditure in Australia, by sex by age (2019 US$)

Age	Male	Female
**<1**	9537.35	8225.14
**1-4 years**	1946.15	1540.07
**5-9 years**	1407.47	1158.21
**10-14 years**	1357.51	1278.25
**15-19 years**	1677.19	2248.60
**20-24 years**	1710.49	2897.45
**25-29 years**	1823.23	3808.70
**30-34 years**	2110.59	4848.75
**35-39 years**	2521.40	4582.59
**40-44 years**	3040.05	4113.00
**45-49 years**	3465.57	4041.90
**50-54 years**	4489.94	4757.85
**55-59 years**	5612.20	5344.07
**60-64 years**	7175.25	6410.48
**65-69 years**	9246.18	8091.24
**70-74 years**	11366.34	9822.93
**75-79 years**	16516.38	12533.93
**80-84 years**	17037.19	14161.53
**85+ years**	19049.62	15568.81

© 2021 Blakely T et al. *JAMA Health Forum.*

eAppendix 4: SARS-CoV-2 parameters

For each monthly cycle, the number of SARS-CoV-2 infections were split into the following categories for all modelled cases, equivalent to all notified and confirmed cases[1]:

A. Asymptomatic
B. Symptomatic, not admitted to hospital
C. Symptomatic, admitted to hospital
D. Symptomatic, admitted to hospital and ICU
E. Die (may come from anyone of B, C and D)

This is slightly different from our previous model [20] as there is now sufficient within-Australia data (i.e. for Victoria, from the Victorian Department of Health and Human Services (Vic DHHS)) to estimate probabilities of hospitalisation, ICU admission and death directly. A large fraction of people dying did not get admitted to ICU, dying on a general ward or in community care – especially elderly people with a do not resuscitate order. We therefore estimated proportions of cases into four mutually exclusive categories (A, B, C and D) for the quantification of morbidity and health expenditure, and one additional category for the quantification of HALYs lost due to death (E).

In this Appendix we describe in order:

- The epidemiological parameters to split each month's SARS-CoV-2 infections into the five above categories.
- The excess health expenditure assigned to each of the three symptomatic SARS-CoV-2 categories (B, C and D).
- The morbidity-loss assigned to each of the three symptomatic SARS-CoV-2 categories (B, C and D).

Epidemiological parametrisation

Supplementary Table 11 (below) shows the number of cases, hospitalisations, ICU admissions and deaths in Victoria. The dates are deliberately different, so that average time lags are allowed for: up to 14 days from notification to death; subsuming 10 days from notification to ICU admission; subsuming 7 days from notification to hospitalisation.

[1] Undetected cases are not modelled explicitly. They exist (to an unknown degree) but assumed to not incur (much) morbidity or cost.

© 2021 Blakely T et al. *JAMA Health Forum*.

eTable 11: Numbers of confirmed SARS-CoV-2 cases, hospitalisations, ICU admission, and deaths in Victoria, Australia

Sex*	Age	Confirmed cases in VIC: 1 Jan to 14 Aug	Hospitalisations (for cases diagnosed between 1 Jan to 20 Aug)	ICU admissions (for cases diagnosed between 1 Jan and 23 Aug)	Deaths (for cases diagnosed between 1 Jan and 14 Aug)
Female	0-9	434	14	0	
	10-19	774	9	1	
	20-29	1,987	74	8	0
	30-39	1,445	83	4	0
	40-49	1,082	81	13	0
	50-59	975	110	21	3
	60-69	542	122	20	5
	70-79	337	162	23	26
	80-89	534	309	5	112
	90+	379	188	1	86
Male	0-9	479	13	2	
	10-19	879	7	1	
	20-29	1,904	42	3	1
	30-39	1,458	54	7	2
	40-49	1,015	99	15	1
	50-59	847	141	36	9
	60-69	518	159	32	13
	70-79	375	179	21	53
	80-89	306	198	8	97
	90+	154	102	0	67
Total		16424	2146	221	475

Supplementary Figure 2 shows the ln odds of hospitalisation, ICU admission and deaths for observed data when the number of events is 5 or more, and from simple predictive logistic regression models on the same data. For the latter regressions, main effects were included for sex and age as a continuous variable, and additional age-dummies due to non-linearity on the ln odds scale for:

- Hospitalisation: 0-9, 10-19, 80-89 and 90+ year olds
- ICU admission: 80-89 and 90+ year olds
- Deaths: 90+ year olds.

© 2021 Blakely T et al. *JAMA Health Forum.*

eFigure 2: Ln odds of hospitalisation, ICU admission, and death for confirmed cases for observed data when number of events >5 and from a logistic regression prediction otherwise

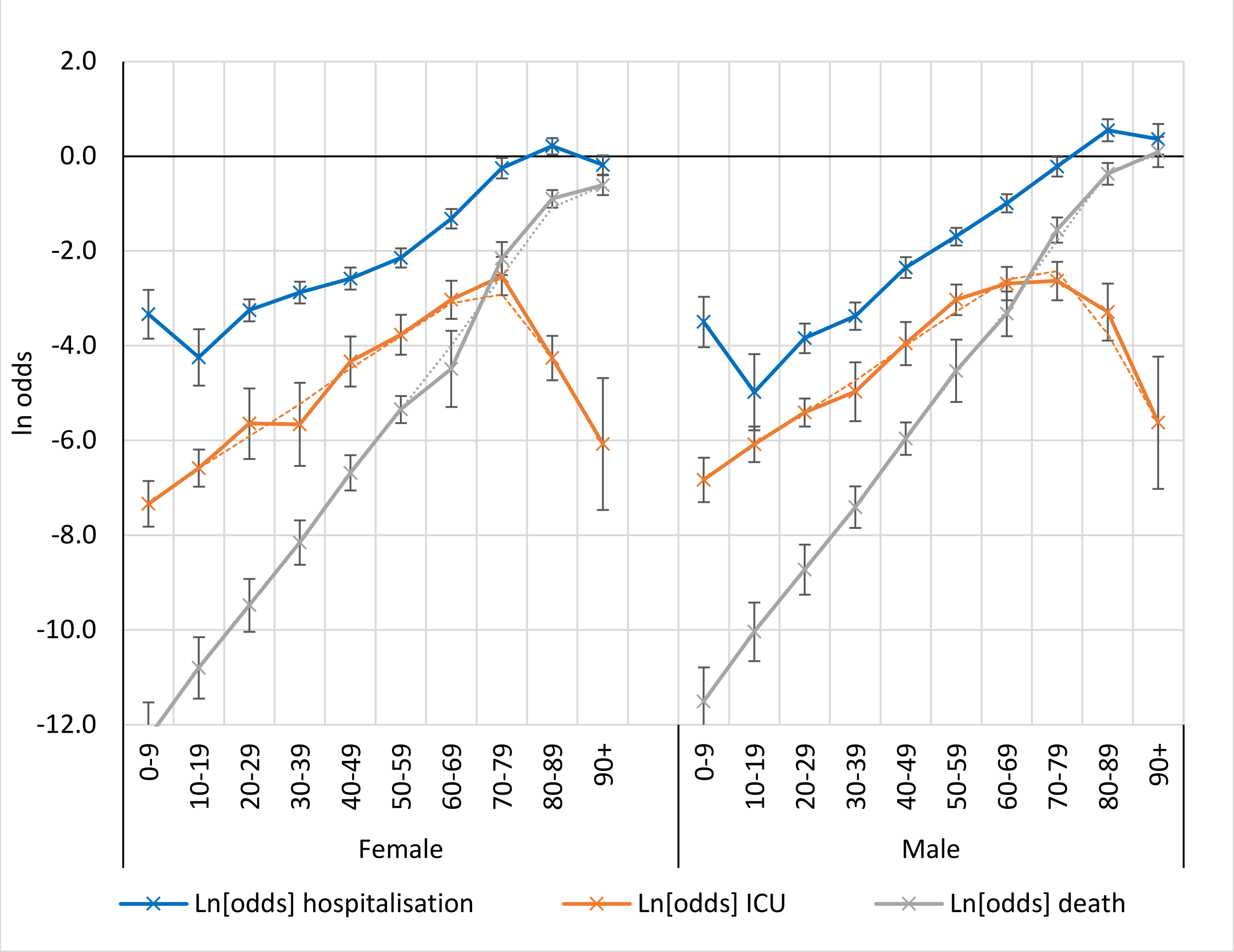

Error bars are 95% confidence intervals

We elected to use the observed ln odds when the number of events was five or more, otherwise use the logistic regression predicted ln odds. These estimates, and their standard errors, are shown in Supplementary Table 12.

Sequentially, the process to estimate the actual disaggregation of SARS-CoV-2 cases (outputted by the ABM) by category of morbidity and mortality was:

- Estimate the monthly number of deaths (E) by sex and age; propagate through the PMSLT increasing mortality rates (no change in morbidity)
- Estimate the number of ICU admissions (D) by sex and age; propagate through the PMSLT increasing morbidity and health expenditure (no change in mortality)
- Estimate the number of hospitalisation admissions (B) by sex and age, subtracting off ICU admissions; propagate through the PMSLT increase morbidity and health expenditure (no change in mortality)
- Estimate number of asymptomatic cases (A).

© 2021 Blakely T et al. *JAMA Health Forum*.

- And finally, estimate the number of symptomatic cases, as the total number of cases, minus (A + C + D + E). And link them to morbidity and health expenditure.

(The estimates of morbidity and excess health expenditure are described in subsequent sections of this Appendix.)

© 2021 Blakely T et al. *JAMA Health Forum.*

eTable 12: ln odds (standard errors) used in simulation modelling

		Hospitalisation		ICU		Death	
**Sex**	**Age**	**ln(odds)**	**s.e. ln(odds)**	**ln(odds)**	**s.e. ln(odds)**	**ln(odds)**	**s.e. ln(odds)**
Female	0-9	-3.335	0.263	-7.339	0.246	-12.276	0.383
	10-19	-4.246	0.304	-6.584	0.200	-10.800	0.331
	20-29	-3.252	0.118	-5.644	0.379	-9.481	0.285
	30-39	-2.874	0.117	-5.660	0.448	-8.156	0.240
	40-49	-2.583	0.119	-4.334	0.269	-6.685	0.190
	50-59	-2.145	0.105	-3.766	0.216	-5.346	0.147
	60-69	-1.321	0.105	-3.027	0.205	-4.491	0.411
	70-79	-0.250	0.110	-2.527	0.208	-2.158	0.179
	80-89	0.211	0.087	-4.260	0.239	-0.898	0.096
	90+	-0.184	0.103	-6.076	0.710	-0.612	0.107
Male	0-9	-3.497	0.271	-6.832	0.239	-11.510	0.366
	10-19	-4.979	0.410	-6.079	0.191	-10.038	0.315
	20-29	-3.843	0.160	-5.408	0.151	-8.727	0.269
	30-39	-3.377	0.147	-4.972	0.317	-7.407	0.224
	40-49	-2.350	0.112	-3.955	0.232	-5.962	0.176
	50-59	-1.694	0.095	-3.027	0.164	-4.530	0.335
	60-69	-0.993	0.099	-2.688	0.180	-3.324	0.240
	70-79	-0.221	0.104	-2.633	0.207	-1.556	0.136
	80-89	0.549	0.119	-3.289	0.307	-0.370	0.116
	90+	0.362	0.165	-5.623	0.712	0.093	0.163

© 2021 Blakely T et al. *JAMA Health Forum*.

eTable 13: Parametrisation of SARS-CoV-2 asymptomatic cases

Age Group	Percentage symptomatic, with 20% of all infections across age asymptomatic		
	Percentage symptomatic	Mean (ln odds scale)	SD
0 to 9	35%	-0.639	0.302
10 to 19	56%	0.235	0.302
20 to 29	75%	1.109	0.302
30 to 39	88%	1.983	0.302
40 to 49	95%	2.857	0.302
50 to 59	98%	3.731	0.302
60 to 69	99%	4.605	0.302
70 to 79	100%	5.479	0.302
80+	100%	6.353	0.302

Morbidity loss by SARS-CoV-2 category

An 'average' incremental morbidity impact due to SARS-CoV-2 for each month was estimated as the weighted sum of the morbidity impact for each of the five symptomatic SARS-CoV-2 categories (weighted by proportionate distribution of SARS-CoV-2 infections by category – which varied iteration to iteration given the uncertainty described above). We measured morbidity impacts using disability rates (DR),[21] according to severity of acute infection (mild, moderate and severe). For ICU admissions, DR were based on severe chronic obstructive pulmonary disease (COPD) to reflect Acute Respiratory Distress Syndrome (ARDS). We assume all survivors return to their baseline health status (pre-SARS-CoV-2) (DR: 0) following a specified recovery period as described below.

Morbidity loss for the four categories of symptomatic SARS-CoV-2 infection include:

- Morbidity for people admitted to ICU, but surviving, assuming a mean duration from symptom onset to recovery of 6 weeks was based on the higher range of the median time from onset to clinical recovery for patients with severe or critical disease.[22] We applied a DR for moderate acute infectious episode for 1 week of 0·051 (0·032–0·074), plus DR for severe acute infection for 2 weeks of 0·133 (0·088–0·190), and ICU admission for 1 week of 0.408 (0.273-0.556), plus a return to baseline health (DR: 0) over 2 weeks (equivalent to 50% probability of ARDS for 2 weeks).
- Morbidity for people admitted to hospital but not requiring ICU, assuming a mean duration from symptom onset to recovery of 4 weeks, based on the lower range of the median time from onset to clinical recovery for patients with severe or critical disease. We applied a DR for moderate acute infectious episode for 1 week of 0·051 (0·032–0·074), plus severe infectious episode for 2 weeks of 0·133 (0·088–0·190), plus return to baseline health (DR: 0) over 1 week (equivalent to 50% probability of severe infectious episode for one week).
- Morbidity for people diagnosed with symptomatic disease but not admitted to hospital, assuming a mean duration from symptom onset to recovery of 2.5 weeks, based on data from the WHO-China Joint Mission report on median time from onset to clinical recovery for mild cases of approximately 2 weeks.[23] We assume half of people with symptomatic disease who are not admitted to hospital have mild symptoms, and the other half have moderate symptoms.[22] Therefore we applied a DR of 0·051 (0·032–0·074) for moderate acute infection for 50% of this group for 2.5 weeks and a DR of 0·006 (0·002–0·012) for mild acute infection for 2.5 weeks for the remaining 50% of this category.

© 2021 Blakely T et al. *JAMA Health Forum*.

The overall morbidity for each SARS-CoV-2 category was calculated as the sum of the symptom severity specific DR multiplied by symptom duration.

© 2021 Blakely T et al. *JAMA Health Forum*.

eTable 14: Disability rate distribution and duration by SARS-CoV-2 category

Category	Symptom severity (health state)	Duration	Disability rate, Mean (95% CI)
Admitted to ICU,	Moderate acute infection	1 week	0.051 (0.032-0.074)
	Severe acute infection	2 weeks	0·133 (0·088–0·190)
	In-ICU (COPD for ARDS)	1 weeks	0.408 (0.273-0.556)
	Return to baseline health	2 weeks	Linearly to 0 from ARDS DR
Admitted to hospital, no ICU	Moderate acute infection	1 week	0.051 (0.032-0.074)
	Severe acute infection	2 weeks	0.133 (0·088–0·190)
	Return to baseline health	1 week	Linearly to 0 from severe acute infection DR
Symptomatic, not admitted	50% Moderate acute infection	2.5 weeks	0.051 (0.032-0.074)
	50% Mild acute infection	2.5 weeks	0·006 (0·002–0·012)

We assumed those who died from SARS-CoV-2 in the community have the same morbidity as ICU deaths.

Excess health expenditure by SARS-CoV-2 category

We estimated the excess health expenditure by SARS-CoV-2 category using an ingredients approach. For each patient category, we modelled the expected patient pathway through the health system, based on available SARS-CoV-2 data from China, Australia, and the UK (Supplementary Tables below). For each patient subgroup category described in the paper, we calculated total health expenditure by first estimating resource use required for a typical patient (e.g., hospital or outpatient visits, or drugs), and multiplying by unit costs for each of the specified resources.

Supplementary Table 15 shows resource use assumptions based on clinical costing data. All patient categories are assumed to have similar patient pathways. Patients who die can come from several of the patient categories below. Current Victorian data has shown that many elderly patients who died as a result of SARS-CoV-2 infection did not receive ICU care. Unfortunately, the data did not allow us to determine exactly what proportion of patients who died accessed ICU care. From the available data, which captures all Victorian SARS-CoV-2 patients up to August 28, we determined that if all possible deaths came from ICU wards, 18% of all deaths (94/513) would have come from an ICU ward. We know that all ICU-admissions will not result in death. We have therefore conservatively estimated that 10% of all patients who die from SARS-CoV-2, have incurred an ICU visit. While it is understood that most COVID-19 deaths in Victoria have occurred in hospital, some deaths have occurred in aged care facilities without being transferred to hospital.[24] As it currently unknown how many patients have died without receiving hospital treatment, we assume that end-of-life care within these facilities will require similar levels of health care as hospitalisation.

The SARS-CoV-2 pandemic is expected to add additional costs to hospital operations, adjusting for complexity of patients and added infection control required including the need for isolation of patients, staff time for proper fitting of personal protective equipment, and enhanced cleaning regiments. As a result, inpatient and ICU hospital costs have been scaled up by 20% to account for these extra costs. This 20% estimate is based on the Coronavirus Aid, Relief and Economic Security (CARES) Act in the United States that provides a 20% add-on payment for COVID-19 patients.[25]We expect that this loading will be a moderate estimate, and likely underestimate the true hospital costs during a pandemic outbreak. [25]Total costs for each patient category described above is found in Supplementary Table 15.

© 2021 Blakely T et al. *JAMA Health Forum*.

eTable 15: Resource use assumptions by SARS-CoV-2 category

Patient Subgroup	Treatment items	Source
**Treated in ICU and survived**	GP Visit<br><br>ER visit<br><br>ICU<br><br>Inpatient (non-ICU)<br><br>Pandemic loading	Assumption that half these patients will have contact with a GP prior to hospitalisation<br>Base fee charged for presenting to an ER<br><br>Cost of ICU day X LOS<br><br>Cost of inpatient day X LOS<br>All hospital costs have 20% loading for pandemic†
**Non-ICU hospitalisation and survived**	GP Visit<br><br>ER visit<br><br>Inpatient<br><br>Pandemic loading	Assume all these patients will have contact with GP prior to hospitalisation<br>Base fee charged for presenting to an ER<br><br>Cost of inpatient day X LOS<br>All hospital costs have 20% loading for pandemic
**Symptomatic case, no hospitalisation and survived**	GP Visit<br><br>Paracetamol	Assume patients have two GP appointments on average (initial visit and follow up appointment)<br>Assume on average all patients will purchase 1 round of paracetamol for symptom reprieve
**Infected case with no symptoms**	No resources	
**Died**	GP Visit<br><br>ER visit<br>ICU<br><br>Inpatient (non-ICU)<br>Pandemic Loading	Assumption that these patients will have 2 contacts with a GP prior to hospitalisation<br><br>Base fee charged for presenting to an ER<br>10% of patients in this category: Cost of ICU day X length of stay (LOS) +Cost of inpatient day X inpatient LOS<br>90% of patients in this category: Cost of inpatient day X LOS<br>All hospital costs have 20% loading for pandemic

† https://revcycleintelligence.com/news/how-much-will-the-covid-19-pandemic-cost-hospitals; accessed 5 May 2020
ER= Emergency Room visit, ICU= Intensive care unit, LOS= Length of stay, GP= General Practitioner

Supplementary Table 16 below shows the input quantities for hospital length of stay across the different patient group categories.

© 2021 Blakely T et al. *JAMA Health Forum*.

eTable 16: Quantity inputs for Hospital (inpatient and ICU) length of stay

Patient Subgroup	Length of Stay	Detail
**Died**:		Can come from ICU, Hospital, or other.
**Inpatient 11 days** (90%)	11 days (total)[26] N=8	90% of patients who die incur no ICU stay: FluCAN estimates people who died from COVID had a median inpatient LOS of 11 days.[26]
**ICU 7 days + 4 days inpatient days** (10%)	8 days.[27]N=54, IQR (4-12)<br>6 days. [28]N=698, IQR (3-9)	Patients who died that entered ICU conservatively estimated at 10%: Median length of ICU days for patients who did not survive. Taken as an estimate between the two data points. Estimate fits within both IQRs. Assume 4 inpatient days on top of ICU LOS (from 11-day FLUCAN estimate).
**ICU Survived:**<br>**ICU 5.5 days**	7 days. [27]N=137, IQR (2-9)<br>4 days).[28]N=355, IQR (2-8)	Median length of ICU days for patients who survived. Taken as an estimate between the two data points. Estimate fits within both IQRs.
**Inpatient 12 days**	12 days.[28]N= 137, IQR (9-15)	Median length inpatient stays for patients who attended ICU and survived. Single point estimate.
**Non-ICU hospitalisation and survived:**<br>**Inpatient 11 days**	11 days. [29]N= 926, IQR (10-13)	Median length inpatient stays for non-severe patients, not needing ICU. Single point estimate.

* Representative of patients up until 5th July 2019. Data was not been updated on LOS for patients who have died, thus may not adequately reflect aged care outbreaks.

Unit costs for each type of health system utilisation (GP visit, emergency room (ER) visit, inpatient bed day, ICU bed day) were estimated from sources in Supplementary Table 17 (below).

eTable 17: Unit costs for healthcare resource use (2019 AUD)

Resource	Source	Detail
Paracetamol<br><br>$6.60	PBS item 10582Y (100 units, 500mg)	Mild cases have symptoms for 2 weeks.[30] Paracetamol recommendation for mild cases. [31]
GP<br>$38.20	MBS item 23. Level B General consultation. Range ($17.50 - $108.85)	MBS telehealth items price match (3, 23, 36, 44 and 91790, 91800, 91802). Range estimates adapted to 2019 values. [32,33]
ER visit<br>$100.38	Data from Manual of Resource Items and Their Associated Costs (Department of Health) [33]	2005 price adjusted to 2019 price. Cost applied for all patients requiring hospitalisation.
Inpatient day $1551	Data from H1N1 outbreak using AR-DRG code. [34]	Total cost of inpatient day post ICU. Also assumed for all non-ICU patients. Adjusted to 2019 value.
ICU day<br>$6331	Micro-costed from H1N1 outbreak. [34]	Total cost per ICU bed-day. Allied health and overheads included. Adjusted to 2019 value

PBS= Pharmaceutical Benefits Scheme, MBS= Medicare Benefits Schedule, AR-DRG= Australian-refined Diagnostic Related Groups, ICU= Intensive Care Unit

eTable 18: Cost per patient category

Patient Category	Total Cost (2019 $AU)	Total Cost (2019 $AU)
Died	24,665	[deaths not costed separately in final PMSLT; rather deaths assumed to have cost of category below from whence they arose]
Treated in ICU	64,238	$44,641
Non-ICU hospitalisation	20,613	$14,324
Symptomatic case, no hospitalisation	83	$57.68
Infected case with no symptoms	0	0

ICU= Intensive care unit

© 2021 Blakely T et al. *JAMA Health Forum.*

eAppendix 5: Road Traffic Crash

COVID-19 restrictions may result in lower road traffic deaths by reducing the number of people leaving their homes. Simultaneously, such restrictions have been linked to higher car and bike usage on roads, compared to public transport use as individuals aim to socially distance. Similarly, lower road congestion may be linked to higher speeds and potentially higher road traffic deaths. To estimate the effect of COVID-19 restrictions on road traffic deaths in Victoria, we obtained COVID-19 mobility data for January to October 2020 from Apple, which describes the activity of users seeking driving routing directions. This data was supplemented by the data on the road traffic deaths on Victorian roads from January through September 2020, from the Australian Bureau of Infrastructure and Transport Research Economics.

eFigure 3: Apple mobility index for Victoria: Driver routing requests (100 = January Baseline)

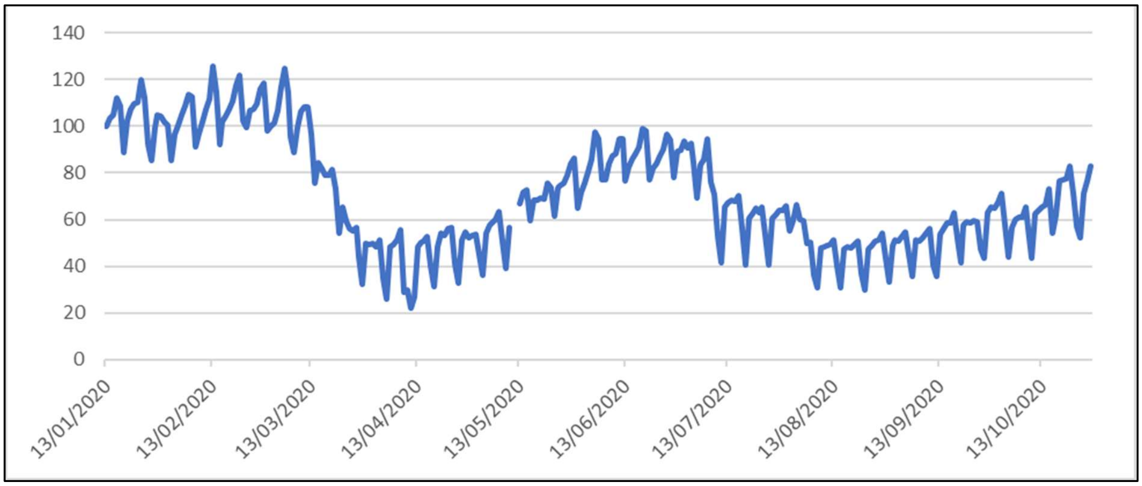

eFigure 4: Victorian Traffic Fatalities by Month (Jan-Sep)

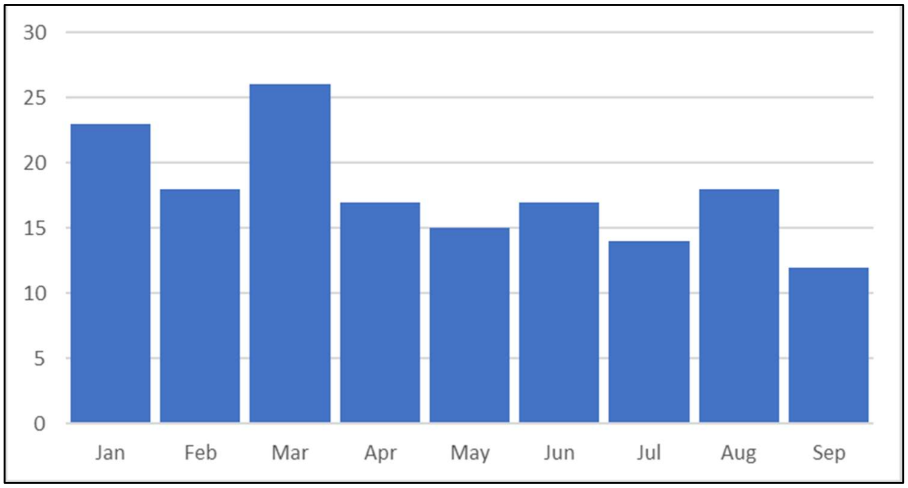

The data was used to calculate the association of a change in the driving mobility index on road traffic deaths using a Poisson regression. A 1-point increase in the mobility index causes a 0.45% increase in road traffic deaths, although the result is not statistically significant. The results of the regression are presented in Supplementary Table 19.

© 2021 Blakely T et al. *JAMA Health Forum.*

eTable 19: Association of Apple Driving Mobility Indices on Road Traffic Deaths

	(1)
	Number of Fatalities
Mobility Index	0.00453
	(1.28)
Day of Week Dummies	YES
Observations	63

t statistics in parentheses

Next, the average Apple driving mobility index was calculated for each stage in VIC through calculating averages using data on restrictions available in Supplementary Table 20. The data was used to calculate the resulting change in road traffic deaths.

eTable 20: Apple mobility driving index for stages 2, 3, and 4 of COVID-19 policy restrictions

**Stage**	**Time**	**Average Apple Mobility Index (100 = Jan 13 Baseline)**	**Change in RTC from Baseline**
4	2nd August – 14th September	47.68	-23.6% (+12.6% to -59.9%)
3	30th Mar – 13th May; 6th July – 2nd August	51.57	-21.93% (+11.3% to -55.5%)
2	13th May – 27th June	80.7	-8.74% (-22.13% to 4.6%)

Stages 1 and 1b are counterfactual. To approximate these mobility indices, we scaled the mobility indices to 0.488 the percent change in GDP (9.42% GDP loss in stage 2 = 19.3% GDP change) from Australian and Victorian Treasuries (See Supplementary Table 9).

eTable 21: Estimated changes in RTC rates for stages 1b and 1

Stage	GDP Change	Mobility Index	RTC
1b	.535b/week = 6.95%	96.6	-1.53% (-3.8% to 0.8%)
1	.5bn/week = 6.5%	96.8	-1.45% (-3.6% to 0.77%)

A limitation of the methodology based on using observational data is that the initial response to Stage 3 restrictions was stronger than later in July, this may be due to the lower national cases of COVID-19 when the second wave. The effect of Stage 3 on RTC cannot be disentangled from this behavior response to the initial wave and may therefore be overestimated.

eReferences

1. Google Inc. COVID-19 Community Mobility Report. United States: Google; 2020.
2. Lauer SA, Grantz KH, Bi Q, et al. The Incubation Period of Coronavirus Disease 2019 (COVID-19) From Publicly Reported Confirmed Cases: Estimation and Application. *Annals of Internal Medicine* 2020; **172**(9): 577-82.
3. Bi Q, Wu Y, Mei S, et al. Epidemiology and transmission of COVID-19 in 391 cases and 1286 of their close contacts in Shenzhen, China: a retrospective cohort study. *Lancet Infect Dis* 2020.
4. Lokuge K, Banks E, Davis S, et al. Exit strategies: optimising feasible surveillance for detection, elimination and ongoing prevention of COVID-19 community transmission. *medRxiv* 2020: 2020.04.19.20071217.
5. He D, Zhao S, Lin Q, et al. The relative transmissibility of asymptomatic COVID-19 infections among close contacts. *International Journal of Infectious Diseases* 2020; **94**: 145-7.
6. Chu DK, Akl EA, Duda S, et al. Physical distancing, face masks, and eye protection to prevent person-to-person transmission of SARS-CoV-2 and COVID-19: a systematic review and meta-analysis. *The Lancet* 2020.
7. Chu DK, Akl EA, Duda S, et al. Physical distancing, face masks, and eye protection to prevent person-to-person transmission of SARS-CoV-2 and COVID-19: a systematic review and meta-analysis. *The Lancet* 2020; **395**(10242): 1973-87.
8. Australian Institute of Health and Welfare. Disease expenditure in Australia. Canberra: AIHW, 2019.
9. Treasury (Victorian Government). Victorian Economic Update, July 2020, 2020.
10. Prime Minister Scott Morrisson. Media release 06 Aug 2020. Canberra: Australian Government; 2020.
11. The Honourable Josh Frydenberg MP (Treasurer of the Commonwelath of Australia), Senator the Honourable Mathias Cormann (Minister for Finance of the Commonwealth of Australia). Economic and Fiscal Update: July 2020. Canbrra: The Treasury, 2020.
12. Australian Bureau of Statistics. Retail Trade, Australia. Monthly and quarterly estimates of turnover and volumes for retail businesses, including store and online sales. August 2020. 2020. https://www.abs.gov.au/statistics/industry/retail-and-wholesale-trade/retail-trade-australia/aug-2020 (accessed 6 Nov 2020 2020).
13. van Baal P, Meltzer D, Brouwer W. Future Costs, Fixed Healthcare Budgets, and the Decision Rules of Cost-Effectiveness Analysis. *Health Econ* 2016; **25**: 237–48.
14. van Baal P, Morton A, Meltzer D, Brouwer W. Future unrelated medical costs need to be considered in cost effectiveness analysis. *Eur J Health Econ* 2019; **20**(1): 1-5.
15. Sanders GD, Neumann PJ, Basu A, et al. Recommendations for conduct, methodological practices, and reporting of cost-effectiveness analyses: Second panel on cost-effectiveness in health and medicine. *JAMA* 2016; **316**(10): 1093-103.
16. Versteegh M, Knies S, Brouwer W. From Good to Better: New Dutch Guidelines for Economic Evaluations in Healthcare. *Pharmacoeconomics* 2016; **34**(11): 1071-4.
17. Australian Bureau of Statistics. Australian Demographic Statistics. 2016.
18. OECD. Evaluating the initial impact of COVID-19 containment measures on economic activity - OECD. https://read.oecd-ilibrary.org/view/?ref=126_126496-evgsi2gmqj&title=Evaluating_the_initial_impact_of_COVID-19_containment_measures_on_economic_activity.
19. Rosenberg B KB, Keene Le L,. Did the 2016 Budget provide enough for health?. : New Zealand Council of Trade Unions; , 2016.
20. Blakely T, Bablani L, Carvalho N, et al. Integrated quantification of the health and economic impacts of differing strategies to control the COVID-19 pandemic. Under review, preprint at SSRN https://ssrn.com/abstract=3605136.

© 2021 Blakely T et al. *JAMA Health Forum.*

21. Salomon JA, Vos T, Hogan DR, et al. Common values in assessing health outcomes from disease and injury: disability weights measurement study for the Global Burden of Disease Study 2010. *Lancet* 2012; **380**(9859): 2129-43.
22. World Health Organization. Coronavirus disease 2019 (COVID-19) Situation Report – 99 WHO, 2020.
23. World Health Organization. Report of the WHO-China Joint Mission on Coronavirus Disease 2019 (COVID-19): World Health Organization, 2020.
24. Services VDoHaH. Transfer protocol for residents in aged care who test positive to coronavirus (COVID-19). In: Services DoHaH, editor.; 2020.
25. Services MLNCfMaM. New Waivers for Inpatient Prospective Payment System (IPPS) Hospitals, Long-Term Care Hospitals (LTCHs), and Inpatient Rehabilitation Facilities (IRFs) due to Provisions of the CARES Act.; 2020.
26. Health Do. COVID-19, Australia: Epidemiology Report 20. In: Health, editor.; 2020.
27. (ICNARC) ICNAaRC. ICNARC report on COVID-19 in critical care 10 April 2020. 2020.
28. Zhou F, Yu T, Du R, et al. Clinical course and risk factors for mortality of adult inpatients with COVID-19 in Wuhan, China: a retrospective cohort study. *The lancet* 2020.
29. Guan W-j, Ni Z-y, Hu Y, et al. Clinical characteristics of coronavirus disease 2019 in China. *New England Journal of Medicine* 2020.
30. World Health Organization T. Report of the WHO-China Joint Mission on Coronavirus Disease 2019 (COVID-19). 2020.
31. Greenhalgh T, Koh GCH, Car J. Covid-19: a remote assessment in primary care. *Bmj* 2020; **368**: m1182.
32. Newall AT, Scuffham PA. Influenza-related disease: the cost to the Australian healthcare system. *Vaccine* 2008; **26**(52): 6818-23.
33. Newall AT, Wood JG, Oudin N, MacIntyre CR. Cost-effectiveness of pharmaceutical-based pandemic influenza mitigation strategies. *Emerg Infect Dis* 2010; **16**(2): 224-30.
34. Higgins AM, Pettila V, Harris AH, et al. The critical care costs of the influenza A/H1N1 2009 pandemic in Australia and New Zealand. *Anaesth Intensive Care* 2011; **39**(3): 384-91.

© 2021 Blakely T et al. *JAMA Health Forum.*
